# Alternative σ Factors Regulate Overlapping as Well as Distinct Stress Response and Metabolic Functions in *Listeria monocytogenes* under Stationary Phase Stress Condition

**DOI:** 10.3390/pathogens10040411

**Published:** 2021-04-01

**Authors:** Renato H. Orsi, Soraya Chaturongakul, Haley F. Oliver, Lalit Ponnala, Ahmed Gaballa, Martin Wiedmann

**Affiliations:** 1 Department of Food Science, Cornell University, Ithaca, NY 14853, USA; rho2@cornell.edu (R.H.O.); hfoliver@purdue.edu (H.F.O.); ag67@cornell.edu (A.G.); 2 Department of Microbiology, Faculty of Science, Mahidol University, Bangkok 10400, Thailand; soraya.cha@mahidol.ac.th; 3 Center of Microbial Genomics (CENMIG), Faculty of Science, Mahidol University, Bangkok 10400, Thailand; 4 Department of Food Science, Purdue University, West Lafayette, IN 47907, USA; 5 Computational Biology Service Unit, Cornell University, Ithaca, NY 14853, USA; lalit.p@gmail.com

**Keywords:** *Listeria monocytogenes*, stress response, alternative sigma factors, SigmaB, SigmaC, SigmaH, SigmaL, RNA-Seq

## Abstract

*Listeria monocytogenes* can regulate and fine-tune gene expression, to adapt to diverse stress conditions encountered during foodborne transmission. To further understand the contributions of alternative sigma (σ) factors to the regulation of *L. monocytogenes* gene expression, RNA-Seq was performed on *L. monocytogenes* strain 10403S and five isogenic mutants (four strains bearing in-frame null mutations in three out of four alternative σ factor genes, ΔCHL, ΔBHL, ΔBCL, and ΔBCH, and one strain bearing null mutations in all four genes, ΔBCHL), grown to stationary phase. Our data showed that 184, 35, 34, and 20 genes were positively regulated by σ^B^, σ^L^, σ^H^, and σ^C^ (posterior probability > 0.9 and Fold Change (FC) > 5.0), respectively. Moreover, σ^B^-dependent genes showed the highest FC (based on comparisons between the ΔCHL and the ΔBCHL strain), with 44 genes showing an FC > 100; only four σ^L^-dependent, and no σ^H^- or σ^C^-dependent genes showed FC >100. While σ^B^-regulated genes identified in this study are involved in stress-associated functions and metabolic pathways, σ^L^ appears to largely regulate genes involved in a few specific metabolic pathways, including positive regulation of operons encoding phosphoenolpyruvate (PEP)-dependent phosphotransferase systems (PTSs). Overall, our data show that (i) σ^B^ and σ^L^ directly and indirectly regulate genes involved in several energy metabolism-related functions; (ii) alternative σ factors are involved in complex regulatory networks and appear to have epistatic effects in stationary phase cells; and (iii) σ^B^ regulates multiple stress response pathways, while σ^L^ and σ^H^ positively regulate a smaller number of specific pathways.

## 1. Introduction

Bacteria may use a diverse set of strategies to survive stress conditions, including a number of mechanisms that act at the level of transcriptional regulation. One important regulatory mechanism in bacteria that enables the transcription of a targeted set of genes under appropriate environmental conditions is mediated through differential associations between various alternative sigma (σ) factors and the core RNA polymerase. Moreover, σ factors are dissociable subunits of prokaryotic RNA polymerase responsible for promoter recognition. The regulon of a single alternative σ factor can include hundreds of transcriptional units; thus, σ factors provide an effective mechanism for simultaneously regulating large numbers of genes under appropriate conditions. Critical phenotypic functions regulated by alternative σ factors range from bacterial sporulation to stress-response systems [1].

*Listeria monocytogenes*, a Gram-positive foodborne pathogen of the Firmicutes family, is the etiological agent of the disease known as listeriosis. As approximately 20% of listeriosis cases result in death in humans, with an estimated annual human death toll of approximately 255–305 in the US alone [2,3,4], this disease is a considerable public health concern. As a foodborne pathogen (with 99% of human illnesses caused by a foodborne route of infection), *L. monocytogenes* is exposed to a diversity of stress conditions in food-associated environments, the food, and the host, where it has to survive passage through the gastrointestinal tract before invasion of epithelial cells in the small intestine and subsequent systemic spread. *L. monocytogenes* has a well-established ability to survive and grow under many conditions that are typically applied to control bacterial populations in foods, such as low pH, low temperature, and high salt conditions [5,6,7].

Regulation of *L. monocytogenes* gene expression in response to different environmental conditions involves a number of transcriptional regulators, including more than 20 two-component regulatory systems, transcriptional repressors such as HrcA and CtsR, the positive regulator of virulence gene PrfA [8], the branched chain amino acid sensor CodY [9], and four alternative σ factors (σ^B^, σ^C^, σ^H^, σ^L^). However, σ^C^, an extracytoplasmic function (ECF) σ factor, is present only in *L. monocytogenes* strains classified as lineage II [10]. Previous transcriptional profiling studies [11,12,13,14,15] have shown that at least some of these alternative σ factors regulate large regulons (e.g., >100 genes positively regulated by σ^B^) and indicate considerable overlap between alternative σ factor regulons, contributing to complex regulatory networks in this organism. Phenotypic studies have also shown that alternative σ factors contribute to resistance to a number of stress conditions (for recent reviews, see References [16,17]). For example, in one study, a *sigL* mutant presented impaired growth relative to the parent strain at low temperature, in the presence of salt, and under lactic acid stress [18]. Phenotypic contributions of σ^C^, σ^H^, and σ^L^ to *L. monocytogenes* stress resistance have been challenging to define, however, and in some studies, no clear effects of deletions in these genes, on different stress-response phenotypes, have been observed [12]. On the other hand, σ^B^ clearly shows robust contributions to *L. monocytogenes* survival or growth under salt stress, acid stress, oxidative stress, and starvation, as supported by a number of studies [11,12,15,19,20,21] and as summarized in recent reviews [16,22]. Importantly, σ^B^ also plays a role in regulating virulence-related functions [17,22,23,24,25,26,27].

Tiling arrays and RNA sequencing (RNA-Seq) approaches have been used to further characterize the *L. monocytogenes* σ^B^ [13,15,28] and σ^H^ [14] regulons, but these approaches have not yet been used to characterize other alternative σ factor regulons in *L. monocytogenes*. In addition, most transcriptional profiling studies performed thus far to characterize *L. monocytogenes* alternative σ factor regulons used single σ factor deletion mutants (e.g., a parent strain and an isogenic *sigB* null mutant); as it has been established in *Bacillus subtilis* [28], this approach will likely not fully define alternative σ factor regulons, due to overlaps between regulons and cooperative effects among alternative σ factors. In addition, two recent publications used a system where σ^B^ [15] or σ^H^ [14] were overexpressed from a rhamnose inducible promoter, in a *L. monocytogenes* null mutant with deletion of all four alternative σ factor genes, to further define the σ^B^ and σ^H^ regulons, respectively. In order to further characterize and compare the regulons for all four *L. monocytogenes* σ factors (σ^B^, σ^H^, σ^C^, and σ^L^), we used a set of *L. monocytogenes* mutant strains that express only one of the four alternative σ factors (from their native promotors), as well as a quadruple mutant expressing only the housekeeping σ factor, σ^A^. The transcriptional profiles of these mutants, grown to stationary phase, were characterized by using an RNA-Seq approach. We reasoned that comparative analyses of four strains that each express a single σ factor from a native promotor would allow for further insights into the contributions of alternative σ factors to regulation of stress response and metabolic functions in *L. monocytogenes* and will specifically allow novel insight into epistatic and additive interactions of σ factors. The data obtained with this approach support the notion that, across bacterial groups, alternative σ factors not only contribute to specific stress response, virulence, and other functions, but also play critical roles in modulating central metabolic functions to facilitate growth and survival under different environmental conditions.

## 2. Materials and Methods

### 2.1. Strains and Growth Conditions

RNA-Seq was performed on (i) the *L. monocytogenes* parent strain 10403S; (ii) four isogenic triple mutants with internal non-polar deletions in three out of four alternative σ factor genes (i.e., ΔCHL [FSL C3-139], ΔBHL [FSL C3-138], ΔBCL [FSL C3-137], and ΔBCH [FSL C3-128]), thus expressing only a single alternative σ factor (i.e., σ^B^, σ^C^, σ^H^, and σ^L^, respectively); and (iii) an isogenic quadruple mutant (i.e., ΔBCHL [FSL C3-135]), which expresses none of the four alternative σ factors (Table 1). Mutants were constructed, using splicing by overlap extension (SOE) PCR and standard allelic exchange mutagenesis, as previously described [29,30]. Growth curves with three biological replicates were carried out and showed that the five mutant strains grow similarly to the parent strain, 10403S in BHI at 37 °C (Supplementary Materials Figure S1). Prior to RNA isolation, bacteria were grown to stationary phase (OD_600_ = 1.0 + 3 h of incubation), in Brain Heart Infusion (BHI) media (BD Difco, Franklin Lakes, NJ, USA), as previously described [13]. Briefly, strains were grown in 5 mL BHI broth, at 37 °C, with agitation (230 rpm) for 15 h. Then, 1% inoculum was transferred to 5 mL of pre-warmed BHI and grown to OD_600_ ~ 0.4. A 1% inoculum was then transferred to a 300 mL nephelo flask (Bellco, Vineland, NJ) containing 50 mL of pre-warmed BHI. This culture was incubated at 37 °C, with agitation, until cells reached stationary phase. Two independent growth replicates and RNA isolations were performed for each strain.

### 2.2. RNA Isolation, Integrity, and Quality Assessment

Cultures grown to stationary phase were treated with RNAProtect bacterial reagent (Qiagen, Valencia, CA, USA), according to the manufacturer’s instructions. Cell pellets were suspended in 5 mL of TRI Reagent (Life Technologies, Gran Island, NY, USA), followed by mechanical disruption (bead-beating with 0.1 mm acid-washed zirconium beads), and RNA extraction, using TRI Reagent, per the manufacturer’s protocol (Life Technologies, Gran Island, NY, USA). Total RNA was incubated with RQ1 DNase (Promega, Madison, WI, USA), in the presence of RNasin (Promega), to remove the remaining DNA. Subsequently, RNA was purified, using two phenol–chloroform extractions and one chloroform extraction, followed by RNA precipitation and re-suspension of the RNA in RNAse free water. UV spectrophotometry (Nanodrop 1000, Wilmington, DE, USA) was used to quantify and assess purity of the RNA, with the 260 nm/280 nm ratio used to assess the presence of proteins in the sample and the 260 nm/230 nm ratio used as a secondary quality measure, to determine the presence of organic contaminants (e.g., Trizol, phenol, Guanidine HCL, or guanidine thiocyanate). Efficacy of the DNase treatment was assessed by TaqMan qPCR analysis of DNA levels of the housekeeping gene *rpoB*; all samples had DNA log copy numbers ≤ 1.5 copies per 10 ng of RNA and C_t_ values > 35 cycles, indicating negligible levels of DNA contamination. RNA integrity was assessed by using the 2100 Bioanalyzer (Agilent, Foster City, CA, USA).

### 2.3. mRNA Enrichment

Removal of 16S and 23S rRNA from total RNA was performed, using the MICROBExpress^TM^ Bacterial mRNA Purification Kit (Life Technologies, Gran Island, NY, USA) according to the manufacturer’s protocol, with the exception that no more than 5 mg total RNA was treated per enrichment reaction. Each RNA sample was divided into multiple aliquots of ≤ 5 mg RNA, which were used for separate enrichment reactions. Enriched mRNA samples were pooled and run on the 2100 Bioanalyzer (Agilent), to confirm reduction of 16S and 23S rRNA prior to preparation of cDNA fragment libraries.

### 2.4. Preparation of cDNA Fragment Libraries and RNA-Seq

For the non-directional runs and the directional runs, the Illumina Genomic DNA Sample Prep kit and the Illumina Directional mRNA-Seq Library Prep kit (Illumina, Inc., San Diego, CA, USA) were used, respectively, according to the manufacturer’s protocol, to fragment RNA followed by phosphatase and polynucleotide kinase (PNK) treatment, ligations of 3′ and 5′ adapters, and reverse transcription of adapter-ligated RNA. Purified libraries were loaded onto independent flow cells; sequencing was carried out by running 32 cycles on the Illumina Genome Analyzer.

### 2.5. RNA-Seq Alignment and Coverage

The 10403S finished genome was used to align Illumina RNA-Seq reads. These alignments were performed, using the Burrows–Wheeler Aligner (BWA), which allowed up to 2 mismatches. Coverage at each base position along the chromosome was calculated by enumerating the number of reads that align to a given base. Coverage files and tables are available at https://doi.org/10.7298/0mjr-6c90 (permanent link).

### 2.6. RNA-Seq Normalization

The RNA-Seq raw output mapped to each annotated gene was normalized by the length of the genes and for the total number of sequenced reads in each run. The normalized coverage is expressed as RPKM (reads per kilobase of gene length per million reads). In order to determine the gene coverages in the replicates where the directional protocol was not used, the proportion of reads mapping to the sense strand in the directional-run replicate was applied to the total coverage in the non-directional replicate. Fold Changes (FCs) were calculated for each gene, as the average normalized coverage between the two replicates for a given triple mutant (i.e., ΔCHL, ΔBHL, ΔBCL, and ΔBCH strains) divided by the average normalized coverage between the two replicates for the ΔBCHL strain. RNA-Seq quantitative data have been shown to correlate well with qPCR data in *L. monocytogenes* [13] and other organisms [31]. Therefore, qPCR confirmation of the RNA-Seq findings was not deemed necessary.

### 2.7. Differential Expression Analysis

The differential expression of genes in different strains was statistically assessed, using the BaySeq method, implemented in the *bayseq* package available from Bioconductor. This package uses a Bayesian approach to simultaneously assess the likelihood of various models, with each representing a possible pattern of expression for a given gene. The total likelihood that a gene is differentially expressed in the presence of a given alternative σ factor was calculated by summing the individual likelihoods of each model in which that gene is differentially expressed in the presence of that alternative σ factor. Genes were considered differentially expressed by a given alternative σ factor (e.g., σ^B^) if they showed a posterior probability (likelihood) PP > 0.90 of being regulated by that σ factor from the BaySeq analysis and an FC ≥ 5.0 (e.g., σ^B^ up-regulated genes) or FC ≤ 0.2 (e.g., σ^B^ down-regulated genes) between the triple mutant expressing that alternative σ factor (e.g., ΔCHL strain) and the quadruple mutant expressing none of the alternative σ factor (i.e., ΔBCHL strain).

### 2.8. Gene Ontology (GO) Enrichment Analysis

Enrichment of gene ontology terms was assessed, using the GOseq package available from Bioconductor. Genes were classified to the lowest possible level, followed by assignment of appropriate parent terms. Enrichment analysis was carried out only for GO terms that included at least five genes.

### 2.9. Identification of Pathways Regulated by Alternative Sigma Factors

Genes directly up-regulated and indirectly up- or down-regulated by σ^B^, σ^C^, σ^H^, and σ^L^ were mapped onto the *L. monocytogenes* 10403S BioCyc database pathway view [32].

### 2.10. Identification of Noncoding RNAs, 5′ and 3′ Transcript Ends, and Putative Promoters

Here, ncRNAs were defined as regions with (i) RNA-Seq coverage that does not overlap with annotated coding sequences or genes encoding rRNAs or tRNAs on the same DNA strand and (ii) defined breakpoints (start and end) not contiguous with a coverage overlapping a coding sequence on the same strand. These regions could represent ncRNAs, anti-sense RNAs, riboswitches, or small RNAs. Putative Rho-independent terminators in the 10403S chromosome were identified, using the program TransTermHP v2.04. Putative terminators mapping to a region where a high coverage transcript ends were manually annotated, while those not mapping to transcript ends were discarded. The 5′ transcript ends were identified manually for each transcriptional unit with high coverage. Promoters for the four alternative σ factors [12,13] as well as for the housekeeping σ factor σ^A^ (consensus sequence: TTGACA-N_17-19_-TATTAT), were manually identified, using the genome browser Artemis [33], by searching the sequences upstream the previously identified 5′ transcript ends of each transcription unit for sequences resembling the known consensus promoter sequences of the five σ factors.

## 3. Results

### 3.1. Transcripts for Genes Involved in Protein Synthesis, Generation of a Free Energy Source in the Form of NADPH, and Stress Response Are Highly Abundant during Stationary Phase Growth

**Figure 1:**
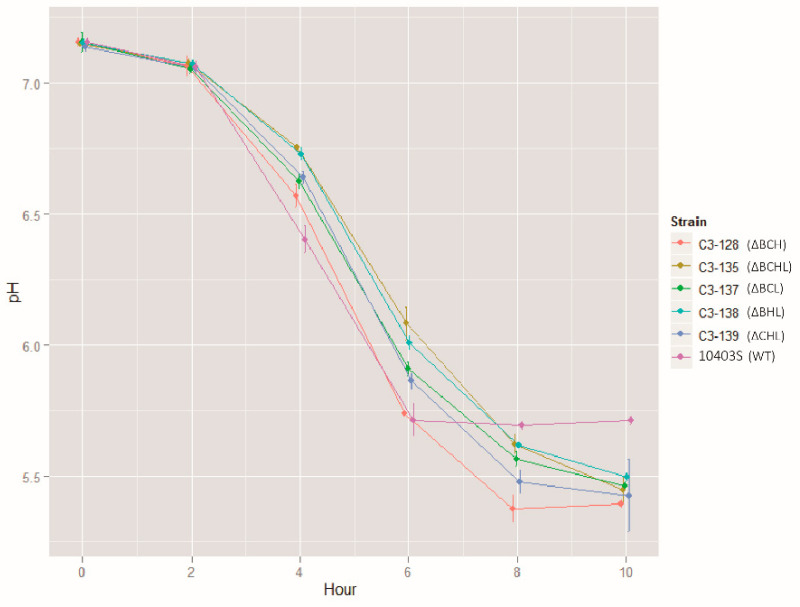
Brain Heart Infusion (BHI) broth pH during growth for 10 h. Measurements were obtained from three independent biological replicates. Vertical bars represent 95% confidence intervals. Although all cultures started with very similar pH values (approximately 7.1), only the parent strain was able to sustain a pH above 5.7 after 10 h of growth. All mutants presented a final pH below 5.5 after 10 h of growth; for all mutant strains, this pH was significantly lower than the pH for the parent strain 10403S (*p* < 0.05; Tukey Honest Significant Difference test).

RNA-Seq experiments were performed, using *L. monocytogenes* grown to stationary phase, as (i) a previous study [12] has shown that stationary phase can be used to study the regulons of all *L. monocytogenes* alternative σ factors, (ii) it has been shown that stationary phase represents a nutrient depletion stress and induces thiol and oxidative stress [34,35], and (iii) because similar conditions may be encountered by *L. monocytogenes* present in food and food associated environments, as well as during host infection. Growth to stationary phase may represent not only a nutrient depletion stress but also a mild acid stress. In our experiments, the broth pH for the parent strain (10403S) stabilized at pH 5.7 after 6 to 10 h of growth (Figure 1), while the ΔCHL, ΔBHL, ΔBCL, ΔBCH, and ΔBCHL mutant strains presented a final pH of 5.4 to 5.5 (after 10 h of growth; Figure 1), which is significantly lower than that of the parent strain at the same time point (adjusted *p*-values < 0.05; Tukey Honest Significant Differences). These findings suggest a reduced ability to mount an acid stress response or an increased production of acidic by-products in the five mutant strains characterized here.

In general, genes that showed the highest transcript levels during stationary phase, in all six strains, encoded proteins with functions involved in response to stress. The most abundant protein-coding transcript in the ΔBCHL mutant was *fri*, which encodes the iron storage protein ferritin, involved in iron binding, virulence, and resistance to oxidative and acid stress [36,37,38,39,40]. The three genes with the next highest transcript levels in this strain encode (i) a protein (Veg) of unknown function that seems to play a role in biofilm stimulation in *Bacillus subtilis* [41], (ii) the flagellin protein (*flaA*), and (iii) an ABC transporter manganese-binding lipoprotein (*mntA*) [42]. Moreover, σ^A^ promoter regions were identified upstream all these genes. In the parent strain 10403S, as well as in the ΔCHL mutant strain, *csbD* (*lmo2158*) was the most abundant transcript. This gene is preceded by an upstream σ^B^-dependent promoter and encodes a small stress-response protein whose function in *L. monocytogenes* and *B. subtilis* is unclear [43]. In the ΔBCH mutant strain, one of the most abundant transcripts is represented by an operon that includes genes encoding D-allose-specific PTS proteins and enzymes for degradation of allose into fructose-6-phosphate; these proteins are predicted to be part of a pathway that allows for utilization of allose as a carbon source for glycolysis.

**Figure 2:**
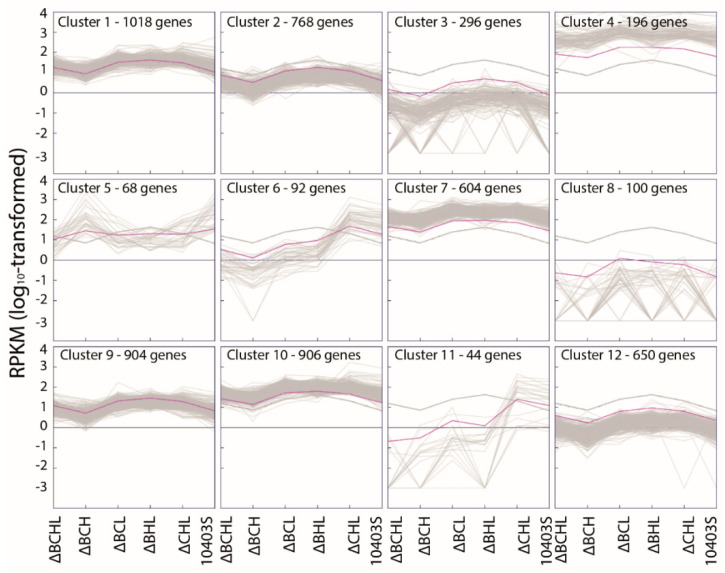
K-means cluster analysis using reads per kilobase of gene length per million reads (RPKM) values. The number of clusters (*n* = 12) was chosen based on figure-of-merit (FOM) analysis, to identify groups of genes that show similar levels of expression (expressed as RPKM) across RNA-Seq experiments. From left to right, each cluster shows the mean log10-transformed RPKM values for the ΔBCHL, ΔBCH, ΔBCL, ΔBHL, and ΔCHL mutant strains and the parent strain 10403S for all genes in a given cluster.

A cluster analysis of the RNA-Seq data for all six strains (see Table 2 for summary statistics) was carried out, using the absolute RNA-Seq coverage expressed as RPKM, which is a measure of the RNA abundance, in stationary phase cells. Cluster analysis of the RPKM expression data resulted in 12 clusters (Figure 2). Three clusters (Clusters 4, 7, and 10) contained 853 genes with high overall expression in all six strains; one or more of these clusters were enriched for genes involved in (i) protein synthesis (i.e., GO terms: structural constituent of ribosome, translation, RNA binding and ribosome, and ribonucleoprotein complex); (ii) iron-sulfur cluster assembly; (iii) primary metabolic process; (iv) nucleobase, nucleoside, nucleotide, and nucleic acid metabolic process; (v) ATP binding; and (vi) response to stress. Two clusters (Clusters 3 and 8) showed overall low expression of 198 genes. Although one of these clusters (Cluster 8) showed no significant enrichment, the other cluster (Cluster 3) was enriched for genes involved in (i) phage assembly and (ii) cobalamin (vitamin B_12_) biosynthesis. The remaining seven clusters contained genes that were differentially expressed in one or more of the triple mutants; a detailed description of the differentially expressed genes, based on a separate cluster analysis, is provided in Section 3.3.

### 3.2. Identification of Transcriptional Units, Putative Promoters, and UTRs

Overall, we identified a total of 1609 putative transcription units, including 546 putative operons, in the *L. monocytogenes* parent strain used here (10403S). A previous study using a tiling array and a different strain of *L. monocytogenes*, EGD-e, had identified 517 operons [44]. The average operon length was 3274 nt, and the longest operon had 23,883 nt. For 500 transcription units, the transcription start site could not be unambiguously identified due to low RNA-Seq coverage in these regions. Therefore, RNA-Seq coverage data allowed the identification of 1109 putative transcription start sites and 5′ untranslated regions (5′ UTR). The average size of the 5′ UTRs was 69 nt; the longest 5′ UTR had 1698 nt (*mogR*). For 57 of the 1109 transcription start sites identified, a putative promoter could not be identified, probably due to high sequence divergence from the known consensus sequences of the five σ factors in *L. monocytogenes*. In addition to 944 putative σ^A^-dependent promoters, we identified 103 putative σ^B^-dependent promoters and five putative σ^H^-dependent promoters.

### 3.3. Clusters of Genes with Similar Patterns of Alternative σ Factor-Dependent Transcript Levels Show Enrichment for Specific Biological Functions

**Figure 3:**
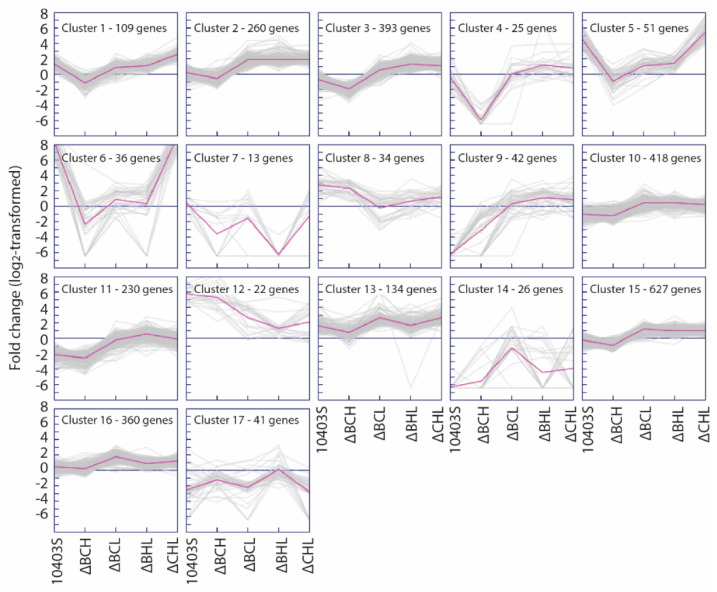
K-means cluster analysis using Fold Change (FC) values (parent or triple mutant strain/ΔBCHL mutant strain). The number of clusters (*n* = 17) was chosen based on figure-of-merit (FOM) analysis to identify groups of genes with similar patterns of expression in the parent strain and each of the ΔBCH, ΔBCL, ΔBHL, and ΔCHL strains in comparison to the ΔBCHL strain. From left to right, each cluster shows the mean log2-transformed FC values for the parent strain, ΔBCH, ΔBHL, ΔBCL, and ΔCHL mutant strains for a given cluster.

To characterize alternative σ factor-dependent transcript levels, we calculated FC differences in transcript levels between parent strain (i.e., 10403S) and each strain expressing only one alternative σ factor (i.e., ΔCHL, ΔBHL, ΔBCL, and ΔBCH) and the strain expressing none of the alternative σ factors (i.e., ΔBCHL mutant strain). Cluster analysis of these FC values identified 17 clusters of genes based on FC patterns, with each cluster representing a group of genes with similar expression patterns in the parent strain and the ΔBCH, ΔBCL, ΔBHL, and ΔCHL strains in comparison to the ΔBCHL strain (Figure 3); eight of these clusters (i.e., Clusters 1, 7, 8, 10, 11, 12, 16, and 17) were enriched for genes involved in specific biological or molecular functions. While Cluster 10 contained 418 genes that appear to be solely σ^A^ dependent (as these genes were typically not differentially expressed), four clusters (Clusters 1, 8, 12, and 16) showed FC patterns that indicated positive regulation by one alternative σ factor, one cluster (Cluster 11) showed FC patterns that indicated negative regulation by one alternative σ factor, and the other two clusters (Clusters 7 and 17) showed more complex FC patterns, indicating regulation by multiple alternative σ factors.

Detailed lists of genes in each cluster can be found in Supplementary Materials Table S1. Briefly, Cluster 1 contained 109 genes that showed FC patterns indicating positive regulation by σ^B^ (genes with high positive differential expression in both the ΔCHL mutant expressing σ^B^ and the parent strain, as compared to the ΔBCHL mutant). Although Cluster 1 was not enriched for any specific GO term, this cluster included genes associated with bile tolerance, such as the genes encoding the well characterized carnitine transporter, OpuC [45], and the bile exclusion system BilE [46]. Cluster 16, which contained 360 genes slightly up-regulated (mean FC of 3.72 across 360 gene included in this cluster) in the ΔBCL mutant expressing σ^H^, was enriched for genes classified into the GO term “SOS response”, such as genes involved in DNA repair (e.g., *recA* and *uvrAB*) [47]. Clusters 8 and 12, both of which included genes with high differential expression (mean FC of 6.09 and 73.28 across 34 and 22 genes, respectively) between the parent strain and the ΔBCH mutant expressing σ^L^ (hence representing genes positively regulated by σ^L^), were each enriched for genes classified into the GO terms “phosphoenolpyruvate-dependent phosphotransferase (PTS) activity”, “carbohydrate transport”, and “signal transduction”. Cluster 12 was also enriched for genes classified into the GO terms “ribulose-phosphate-3-epimerase activity” and “hydrogen:sugar symporter activity”, suggesting an important role of σ^L^ in sugar transport and metabolism [48]. Cluster 11 contained genes that were generally down-regulated in both the parent strain (mean FC of 0.26 across 230 genes) and the ΔBCH mutant expressing σ^L^ (representing genes down-regulated by σ^L^; mean FC of 0.18 across 230 genes). This cluster was enriched for genes classified into the GO terms “structural molecule activity”, such as genes encoding ribosomal proteins, and “flagellum”. Cluster 7 contained 13 genes down-regulated by both σ^L^ (mean FC of 0.29) and σ^C^ (mean FC of 0.01), suggesting that σ^L^ and σ^C^ may repress the expression of these genes; this cluster was enriched for genes classified into the GO terms “response to extracellular stimulus” and “establishment of competence for transformation”. Cluster 17, which included 41 genes down-regulated in the parent strain (mean FC of 0.51) and/or the ΔCHL (mean FC of 0.26) and ΔBCL (mean FC of 0.42) mutants expressing σ^B^ and σ^H^, respectively, was enriched for genes classified into the GO terms “phage assembly” and “antigenic variation”. Cluster 10, which included 418 σ^A^-dependent genes, was enriched for genes classified into the GO terms “translation”, “ribonucleoprotein complex”, “alcohol catabolic process”, “cell motility”, and “chemotaxis”.

### 3.4. Differential Expression Analysis Reveals a Large Regulon Positively Regulated by σ^B^ as Well as a Large Regulon Negatively Regulated by σ^L^

**Figure 4:**
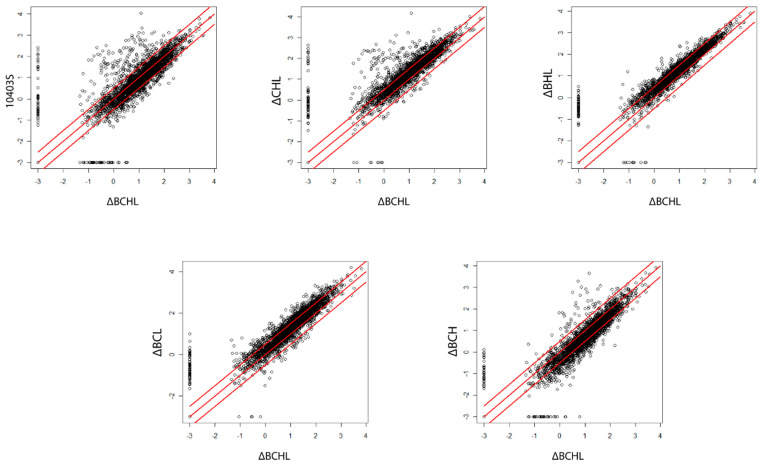
Scatter plot of RPKM data for each gene, comparing the parent strain and the different strains expressing a single sigma factor against the ΔBCHL strain. Centered diagonal red line shows a Fold Change (FC) = 1. Upper and lower diagonal red lines represent FC = 5.0 and FC = 0.2, respectively.

In addition to the cluster analyses detailed above (Section 3.3), we also identified individual genes that showed evidence for being regulated by a given alternative σ factor, as supported by (i) a posterior probability (PP) > 0.90 of being regulated by a given σ factor and (ii) an FC ≥ 5.0 (for identifying genes up-regulated by an alternative σ factor; Supplementary Materials Table S2) or ≤ 0.2 (for identifying genes down-regulated by an alternative σ factor; Supplementary Materials Table S3) between the ΔCHL, ΔBHL, ΔBCL, or ΔBCH mutants (which express σ^B^, σ^C^, σ^H^, or σ^L^, respectively) and the ΔBCHL mutant. As discussed in detail below, the ΔCHL mutant strain, expressing only σ^B^, showed the largest number of up-regulated genes (184 genes), followed by the ΔBCH, ΔBCL, and ΔBHL strains, expressing only σ^L^, only σ^H^, and only σ^C^ (35, 34, and 20 up-regulated genes) (Figure 4). The ΔCHL and ΔBCH mutants also presented the highest number of genes, with FC > 100 (44 and 4 genes, respectively).

### 3.5. σ^B^ Directly Regulates a Large Number of Genes Involved in a Variety of Different Pathways

In addition to the 184 genes found to be up-regulated by σ^B^, five genes, which are all associated with phages, were down-regulated in the strain expressing σ^B^. Among the 184 up-regulated genes, 112 were preceded by a putative σ^B^-dependent promoter (either directly upstream or upstream of the overall operon), suggesting direct regulation by σ^B^. Based on prediction of metabolic pathways for strain 10403S [32], genes up-regulated by σ^B^ (see Supplementary Materials Table S2) encode proteins involved in pathways for cell structure biosynthesis, amino acid biosynthesis (i.e., tryptophan and arginine), biosynthesis of cofactors, prosthetic groups, and electric carriers (i.e., tetrahydrofolate, glutathione, flavin, pyridoxal 5′-phosphate, heptaprenyl diphosphate, nicotinamide adenine dinucleotide, and folate), gluconeogenesis, biosynthesis of fatty acids and lipids (i.e., palmitate, estearate, phosphatidyl glycerol, cardiolipin, and fatty acid elongation), *de novo* biosynthesis of pyrimidine deoxyribonucleotides, fermentation, carbohydrates degradation, aldehyde degradation, amino acid degradation, carboxylates degradation, amine and polyamine degradation, alcohol degradation, and glycolysis. Genes up-regulated by σ^B^ also included four virulence-associated internalin genes (i.e., *inlA*, *inlB*, *inlC2,* and *inlD*), and several genes previously reported as involved in stress response (e.g., *clpC*, *clpX*, *opucABCD*, *gadD3*, *arcA*, *ltrC*, *uspL*-*3*, and *bsh*). Among the stress response genes, several are specifically involved in acid stress response, such as *bsh*, which encodes for the bile salt hydrolase; *uspl-1*, which has been shown to provide acid stress protection to *L. monocytogenes* [49]; *argE*, which participates in arginine biosynthesis from glutamate; and *arcA*, which is directly involved in the arginine deiminase system of acid tolerance. Moreover, a putative acyltransferase (LMRG_00285) showed a 238 FC increase when σ^B^ was expressed. A *Staphylococcus aureus* homolog to this gene was found to be overexpressed (although not significantly) in the presence of paracetic acid, a disinfectant [50]. Furthermore, *opuCABCD*, which encodes for the carnitine ABC transporter involved in osmotolerance, was significantly over-abundant in the presence of σ^B^ (as also previously shown by References [51,52]); this operon has been shown to also contribute to bile acid tolerance [46]. In stationary phase, σ^B^ thus contributes to regulation of a large number of genes involved in many different metabolic pathways, including transport and metabolism of sugars and other macromolecules, virulence, and stress response.

### 3.6. While a Considerable Number of Genes Encoding Sugar Transport and/or Catabolism Are Upregulated in the Strain Expressing σ^L^, None Are Preceded by an Identifiable σ^L^-Dependent Promotor

**Figure 5:**
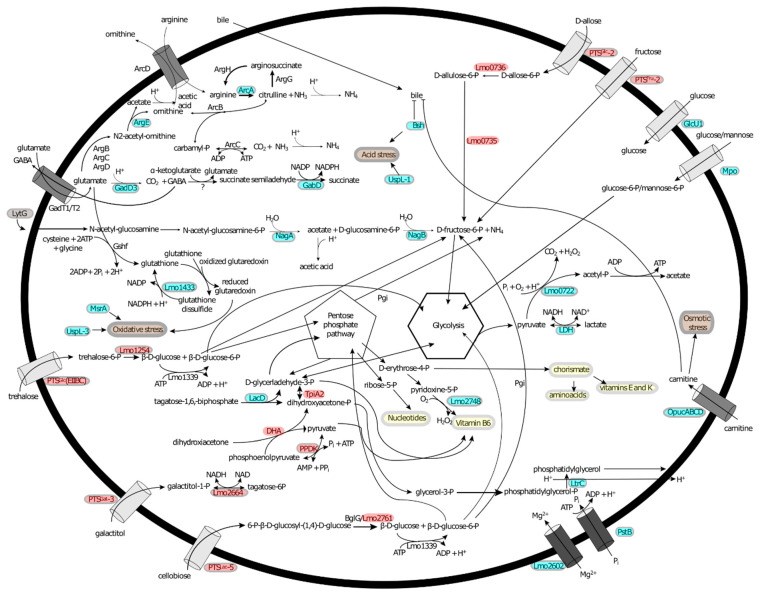
Metabolic map showing major pathways positively regulated by alternative σ factors, based on the findings reported here. Proteins color-coded in red are encoded by genes up-regulated by σ^L^, while proteins in blue are encoded by genes up-regulated by σ^B^, and LytG (color-coded in gray) is encoded by a gene up-regulated by σ^H^ (upregulation always was identified in stationary phase cells). Proteins important to understand the pathways are also shown, but they are not color-coded if their expression was not found to be regulated by any of the four alternative σ factors. Amino acid and carnitine transporters are color-coded in gray, sugar transporters are color-coded in light gray, and Mg^2+^ and P_i_ transporters are color-coded in dark gray. Specific stress types are shown in brown, and proteins known to be involved in a specific stress but with a still unknown mechanism are associated to the stress by arrows. Biosynthesis of nucleotides; vitamins B_6_, E, and K; chorismate; and amino acids are highlighted in yellow, and their precursor metabolites are associated by arrows.

**Figure 6:**
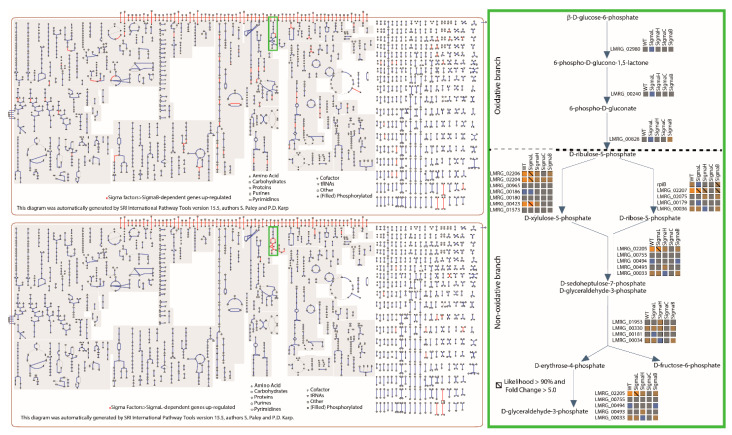
Cellular overview highlighting *L. monocytogenes* strain 10403S pathways regulated by σ^B^ and σ^L^ (**left panels**) and specifically highlighting Pentose Phosphate Pathway (PPP) components regulated by alternative sigma factors (**right panel**). Overview was created with the software Pathway Tools. Top-left panel: Enzymes encoded by genes with expression up-regulated by σ^B^ are highlighted. Bottom-left panel: Enzymes encoded by genes with expression up-regulated by σ^L^ are highlighted. Right panel: The oxidative (top) and non-oxidative (bottom) branches of the PPP are shown. For each enzyme, a colored square shows the level of FC between each strain (on top of the squares) and the *L. monocytogenes* ΔBCHL strain. Blue indicates negative FC (higher expression in ΔBCHL), yellow indicates a positive FC, and gray indicates similar expression levels between the strains. No genes involved in the oxidative branch are significantly regulated by any of the four alternative σ factors. Enzymes encoded by genes significantly up-regulated by σ^L^ and/or σ^B^ have diagonal lines on their expression profiles.

In this study, 35 genes showed evidence for being up-regulated by σ^L^ (Supplementary Materials Table S2 and Figure S2), while 180 genes showed evidence for being down-regulated by σ^L^ (Supplementary Materials Table S3). Unlike σ^B^, which appears to regulate genes involved in a number of different pathways, σ^L^-dependent genes are mainly involved in a few pathways. Specifically, all 35 genes identified as being up-regulated by σ^L^ appear to be involved directly or indirectly (through transcription regulation) in sugar transport and/or catabolism. These 35 genes are clustered into eight operons; only one of these 35 genes, encoding a transcriptional factor similar to LacI (LMRG_00422), is transcribed monocistronically. No σ^L^-dependent promoter was found upstream of these transcription units, suggesting indirect regulation by σ^L^. Moreover, σ^L^ was specifically found to up-regulate PTS-encoding genes involved in the transport of allose, fructose, trehalose, galactitol, and cellobiose, as well as genes involved in the metabolism of these sugars to fructose-6-phosphate (F6P), glucose-6-phosphate (G6P), and glyceraldehyde-phosphate (GAP) (Supplementary Materials Table S2; Figure 5). Additionally, σ^L^ was also found to up-regulate genes involved in all reactions of the non-oxidative branch of the pentose phosphate pathway (PPP) (i.e., ribose-6-phosphate isomerase, ribulose-phosphate-3-epimerase, transketolase, and transaldolase) (Figure 6). Furthermore, σ^L^ was found to up-regulate two genes (*ccpN* and *yqfL*) that had been reported to be involved in glycolysis/glyconeogenesis regulation in *B. subtilis* (Supplementary Materials Table S2) [53,54]. In *B. subtilis*, CcpN represses the transcription of two genes, *gapB* and *pckA*, which encode enzymes responsible for non-reversible reactions during gluconeogenesis [55]. While *L. monocytogenes* lacks genes homologous to *gapB* and *pckA*, *L. monocytogenes* has a gene (*gap*) that encodes a glyceradehyde 3-phosphate dehydrogenase, which catalyzes, reversibly, the same reactions facilitated by the *gapA* and *gapB* gene products in *B. subtilis*. We, however, found no evidence for σ^L^ dependent transcription of *gap* (FC = 1.02). *L. monocytogenes*, however, has an optional route to convert malate into pyruvate through a malate dehydrogenase encoded by LMRG_01062, a gene that is significantly down-regulated in the presence of σ^L^ and may be a possible target of CcpN in *L. monocytogenes*. Thus, σ^L^ could be responsible for regulating the early stages of gluconeogenesis, since malate dehydrogenase is the first enzyme in the *L. monocytogenes* gluconeogenesis pathway. The 180 genes identified as being down-regulated by σ^L^ appear to encode proteins involved in various functions, such as protein biosynthesis, motility (flagellar biosynthesis), sugar transport and metabolism (including a number of ABC transporters), and cobalamin (i.e., vitamin B_12_) biosynthesis. Overall, σ^L^ seems to play a major role in sugar acquisition and energy generation in late stationary phase (which likely represents nutrient-depletion stress).

### 3.7. The σ^H^ Regulon Overlaps with other Alternative σ Factor Regulons, but Solely Regulates Genes Associated with DNA Transformation/Competence

Overall, 34 genes showed higher transcript levels in the presence of σ^H^, indicating up-regulation by σ^H^ (Supplementary Materials Table S2), while five genes showed evidence for being down-regulated by σ^H^ (Supplementary Materials Table S2). Five σ^H^ consensus promoter sequences were identified upstream of four genes and one operon with at least one σ^H^-up-regulated gene. The σ^H^-dependent genes with upstream σ^H^ consensus promoters included the following: (i) *comEA* and *coiA*, both encoding proteins involved in establishment of competence; (ii) *rpoD*, encoding the vegetative σ factor RpoD (i.e., σ^A^); (iii) *lytG* encoding the glucosaminidase LytG, which, in *B. subtilis*, is responsible for peptidoglycan structural determination during vegetative growth and is also involved in chemotaxis, motility and cell division [56]; and (iv) an operon encoding three hypothetical proteins (one of them found here to be significantly up-regulated by σ^H^) and two proteins involved in oxidative stress (MsrA and MsrB, neither significantly up-regulated by σ^H^). Moreover, σ^H^ was also found to positively regulate four genes involved in sugar transport and metabolism (Supplementary Materials Table S2); however, these genes were not preceded by a putative σ^H^-dependent promotor, suggesting indirect regulation. Interestingly, four of the five phage-associated genes down-regulated by σ^B^ were also found to be negatively regulated by σ^H^.

### 3.8. σ^C^ Largely Regulates Genes That Have Not yet been Assigned Specific Functions and Has a Regulon That Shows Considerable Overlap with the Regulons for other Alternative σ Factors

Overall, 19 genes showed higher transcript levels in the presence of σ^C^, indicating up-regulation by σ^C^ (none of these genes were preceded by a putative σ^C^ promoter); no genes showed evidence for being down-regulated by σ^C^. The maximum FC observed among σ^C^ up-regulated genes was 14.5. Together with the low number of σ^C^ -regulated genes identified, this suggests a limited role for σ^C^ during stationary phase. Overall, 12 of the 19 genes up-regulated by σ^C^ are annotated as encoding hypothetical proteins or proteins with unknown functions (see Supplementary Materials Table S2).

### 3.9. A Number of Genes Presented Complex Expression Patterns Suggesting Epistatic and Additive Effects among Alternative σ Factors

In order to identify genes with complex expression patterns (e.g., genes co-regulated by more than one alternative σ factor), indicating interaction among σ factors that alter the regulation of a given gene, the average FCs between each of the ΔCHL, ΔBHL, ΔBCL, and ΔBCH strains and the ΔBCHL strain were analyzed (Table 3). We specifically identified (i) genes showing higher transcript levels (FC > 5.0) in the ΔCHL, ΔBHL, ΔBCL, ΔBCH strains compared to the ΔBCHL strain, but similar transcript levels (0.5 < FC < 2.0) in the parent strain and the ΔBCHL strain (indicating negative epistatic effects), (ii) genes showing higher transcript levels (FC > 5.0) in the ΔCHL, ΔBHL, ΔBCL, and ΔBCH strains, compared to the ΔBCHL strain, but lower transcript levels (FC < 0.5) in the parent strain compared to the ΔBCHL strain (indicating strong negative epistatic effects), and (iii) genes showing higher transcript levels (FC > 5) in at least two of the ΔCHL, ΔBHL, ΔBCL, and ΔBCH strains, compared to the ΔBCHL strain, and even higher transcript levels in the parent strain compared to the ΔBCHL strain (indicating regulation with additive effects). A total of 173 genes showed a negative epistatic effect with the pattern described in (i). Another 18 genes showed a strong negative epistatic effect as described in (ii), suggesting that although these genes may be up-regulated by a single alternative σ factor, the co-expression of other alternative σ factors down-regulates the expression of these genes. Among these 18 genes with strong negative epistatic effect, nine showed FC < 0.2 in the parent strain while showing an FC > 5 in at least one of the ΔCHL, ΔBHL, ΔBCL, and ΔBCH strains. Moreover, eight genes showed patterns suggesting an additive effect of multiple σ factors.

Genes with evidence for negative epistatic effects included genes involved in sugar transport and metabolism (Table 3 and Supplementary Materials Table S4). For example, genes in an operon encoding a galactitol-specific PTS system (LMRG_01244-LMRG_01250) presented transcriptional patterns suggesting a complex regulatory network involving different σ factors. Five out of seven genes in this operon present a pattern of higher transcript levels in the ΔBCL (8.9 < FC < 20.0) and ΔBHL (10.0 < FC < 33.0) strains, as compared to the ΔBCHL strain, but lower transcript levels in the parent strain (0.2 < FC < 0.6), as compared to the ΔBCHL strain. This pattern suggests that simultaneous expression of multiple alternative σ factors may down-regulate the expression of this operon, while the expression of σ^H^ or σ^C^ alone up-regulates the expression of this operon. Other genes with relevant biological functions and patterns of expression that may suggest epistatic interactions among alternative σ factors, but that did not meet our predefined criteria, included LMRG_01332 (*lmo1634*), which encodes for an alcohol acetaldehyde dehydrogenase known as the *Listeria* adhesion protein, LAP [57,58]. This gene presented an FC = 54.1 in the mutant expressing σ^H^ but an FC < 8.0 in all other strains, including the parent strain, where an FC = 2.1 was observed. LMRG_02638 (*lmo0216*), which encodes for a putative ribosome-associated heat shock protein also presented a pattern of high Fold Change in the mutant expressing σ^H^ (FC = 22.7) but not in the parent strain (FC = 3.2). This gene was also highly expressed in the mutant expressing σ^B^ (FC = 11.1), suggesting a complex transcriptional regulatory network.

Only two transcription units presented a pattern suggesting additive effect involving alternative σ factors. One transcription unit with evidence for an additive effect involving σ^B^, σ^H^, and σ^C^ contained only one monocistronically expressed gene (*lmo2066*) encoding a putative protein with unknown function. This gene presented a high positive FC (11.1 < FC < 15.3) in the ΔCHL, ΔBCL, and ΔBHL strains and an even higher FC level in the parent strain (FC = 32.4). The other transcription unit presented an additive effect involving σ^B^, σ^H^, and σ^L^ and contained seven genes (*lmo2662–lmo2668*) encoding proteins involved in sugar transport and metabolism. All genes in this operon presented a high positive FC (8.3 < FC < 72.7) in the ΔCHL, ΔBCL, and ΔBCH strains. However, the parent strain presented FC levels (30.7 < FC < 94.8) that, for each individual gene, were higher than those observed in any of the individual triple mutants. Besides these examples, one gene, LMRG_00095 (*lmo0402*), presented a very high FC in the parent strain (FC = 42.6) but an FC < 5.0 in each of the ΔCHL, ΔBHL, ΔBCL, and ΔBCH strains (Supplementary Materials Figure S2), suggesting that its expression is dependent on at least two of the alternative σ factors. This gene encodes an antiterminator transcription factor of the BglG family [59] and is the last gene in a large operon that includes genes involved in fructose-specific PTS-dependent transport, suggesting that it may be involved in sugar transport and metabolism, a biological function that seems to be regulated by at least two alternative σ factors in *L. monocytogenes*, σ^B^ and σ^L^, as described in Section 3.6, above.

### 3.10. σ^B^ and σ^L^ Regulate Genes That Appear to Represent an Energy Metabolism Network

Several genes involved in sugar transport and metabolism were found to be positively regulated by σ^L^ and σ^B^ (Supplementary Materials Table S5). However, while σ^B^ up-regulates genes involved in the transport of glucose (i.e., *glcU1* and *mpoACD*), σ^L^ up-regulates genes involved in transport and metabolism of alternative sugars, such as galactitol, lactose, and fructose. This difference may suggest a complementary role of σ^L^ in sugar acquisition when extracellular glucose concentration is low. Interestingly, σ^B^ also positively regulates genes encoding enzymes (e.g., LacD) that provide substrates (e.g., glyceraldehyde-3P and dihydroxyacetone-P) for enzymes encoded by genes positively regulated by σ^L^ (e.g., TpiA2), suggesting a metabolic network regulated by σ^B^ and σ^L^ under starvation stress (Figure 5).

### 3.11. Five of More Than 100 ncRNAs Identified Are σ^B^ Dependent

A total of 102 noncoding RNAs (ncRNAs) were identified and annotated in this study (Supplementary Materials Table S6 and Figures S3–S5). While none of these ncRNAs showed evidence for σ^C^ or σ^L^-dependent positive regulation, seven (including two overlapping ncRNAs) and one ncRNAs showed evidence for being positively regulated by σ^B^ and σ^H^, respectively. Three σ^B^-dependent ncRNAs were preceded by a putative σ^B^-dependent promoter; no σ^H^-dependent promoter could be identified upstream the σ^H^-dependent ncRNA transcript. The three differentially regulated ncRNAs with σ^B^-dependent promoters include two previously described σ^B^-dependent ncRNAs, *sbrA* [60] and *rli47* (i.e., *sbrE*) [13,44]. Previous studies have suggested that *sbrE* plays a role in *L. monocytogenes*’ response to acid stress [61] and in restricting growth under harsh conditions [62], and have shown that it is highly induced in the intestine [44] and inside macrophages [63]. The third ncRNA with a σ^B^-dependent promoter has been previously described as *rli33* [44]. This ncRNA is located between two genes (LMRG_00359 and LMRG_00360), both encoding hypothetical proteins, and had previously been shown to be less abundant in an *L. monocytogenes* Δ*sigB* mutant [44]; a mutant strains lacking *rli33* had also previously been shown to be attenuated for murine infection [63]. A σ^B^-dependent promoter was also identified upstream of *rli70*-*2*, a ncRNA not significantly up-regulated by σ^B^ in our analysis (Supplementary Materials Figure S3), probably due to a strong downstream σ^A^-dependent promoter transcribing a shorter version of the ncRNA previously designated *rli70* [13]. This ncRNA, a guanine riboswitch [64], is located upstream of a large operon with five genes that encode enzymes involved in glycolysis and gluconeogenesis and a putative transcriptional regulator similar to CggR, a regulator of central glycolytic genes. Therefore, it is possible that σ^B^ indirectly regulates these two pathways (glycolysis and gluconeogenesis) post-transcriptionally through *rli70-2*. Another σ^B^-dependent promoter was found upstream of an anti-sense ncRNA overlapping a gene similar to the drug/sodium antiporter-encoding gene *yisQ* from *B. subtilis* [65]. Because this anti-sense ncRNA was manually identified post-analysis, no formal statistical analysis was carried to assesses whether it was significantly up-regulated by σ^B^.

### 3.12. Both σ^B^ and σ^H^ Regulate the Transcription of Long 5′ UTRs

We identified six σ^B^-dependent promoters that resulted in long (>100 bp) 5′ UTRs; four of these genes also showed evidence for higher transcript levels in the ΔCHL strain, as compared to the ΔBCHL strain. The six σ^B^-dependent long 5′ UTRs identified include two that represent long partially overlapping 5′ UTRs present on opposite DNA strands (see Supplementary Materials Figure S7). One of these long 5′ UTRs (525 bp) overlaps a gene (LMRG_00335) encoding a putative magnesium and cobalt transporter similar to CorA [66], while the long 5′ UTR (394 bp) on the opposite strand partially overlaps LMRG_00334, which encodes a hypothetical protein. We also identified a previously described σ^B^-dependent long 5′ UTR upstream of *mogR* [44], which encodes a motility regulator; this 5′ UTR overlaps three flagella genes (*fliN*, *fliP* and *fliQ*) in the opposite strand as well as their 5′ UTR and promoter region. Another long σ^B^-dependent 5′ UTR is located upstream of LMRG_01561, which encodes a hypothetical protein. This 5′ UTR overlaps *comK*, which is located in the opposite strand, as well as the *comK* 5′ UTR and transcription start site, suggesting that σ^B^ may indirectly regulate expression of *comK*, which is considered the major regulator of competence in *B. subtilis* [67]; the *L. monocytogenes* 10403S *comK* gene is interrupted by a prophage insertion and, therefore, is likely to be non-functional while the phage stays lysogenic. Another long σ^B^-dependent 5′ UTR results from a σ^B^-dependent promoter identified in the middle of the coding region of *pstB*, which encodes an ATP-binding subunit of the phosphate ABC transporter. This promoter transcribes the second half of *pstB*, as well as the complete coding region of a gene encoding a putative phosphate transport system regulatory protein similar to PhoU; a start codon preceded by a consensus ribosome binding sequence can be found 56 bp downstream of the transcription start site associated with this internal σ^B^-dependent promoter; σ^B^ may thus be involved in regulation of phosphate acquisition. Another σ^B^-dependent promoter resulting in a long 5′ UTR was identified in front of LMRG_01737, a gene encoding a putative negative regulator of σ^L^, similar to *B. subtilis* Hpf [68,69]. In *B. subtilis*, *hpf* is under the dual control of σ^B^, which activates *hpf* transcription under glucose starvation, and σ^H^, which activates transcription under amino acid depletion stress [68] and is mainly required for ribosome dimerization in stationary phase [69]. In *L. monocytogenes*, we identified a σ^A^-dependent promoter upstream LMRG_01737, which induces a very strong transcription of this operon that masks transcription originating from the downstream σ^B^-dependent promoter. No clear role has been defined for this 5′ UTR to date.

The long σ^H^-dependent 5′ UTR identified here is upstream of an operon that includes *msrA*, *msrB*, and a hypothetical protein encoding gene; this operon also includes two upstream σ^A^-dependent promoters. The σ^H^-dependent promoter is the most upstream of the three promoters and overlaps with a gene predicted to encode for a hemolysin-III-like protein in the opposite strand. Although the function of this hemolysin-III-like protein has not been demonstrated in *L. monocytogenes,* it is a transmembrane protein. The overlap between the 5′ UTRs of this hemolysin-III-like encoding gene and the σ^H^-derived promoter on opposite strand may result in post-transcriptional σ^H^-dependent regulation of this hemolysin-III-like protein. No σ^C^- and σ^L^-dependent 5′ UTRs with a length of >100 nt were identified.

## 4. Discussion

In this study, we assessed the transcription profiles of a parent strain (i.e., 10403S) and four *L. monocytogenes* isogenic mutant strains (i.e., ΔCHL, ΔBHL, ΔBCL, and ΔBCH) expressing only one of the four alternative σ factors and compared them against the transcription profile of an isogenic mutant strain (ΔBCHL) expressing none of the alternative σ factors, grown to stationary phase. Differential expression analysis showed that σ^B^ positively regulated the greatest number of genes among the alternative σ factors, while σ^L^ affected transcript level of a greater number of genes overall, accounting for both genes with increased and decreased transcript levels. In addition to considerable σ factors regulon overlap, we also found strong evidence for epistatic and additive interactions among the alternative σ factors evaluated, further supporting that *L. monocytogenes* stress response involves complex regulatory networks. Initial identification of these networks provides an important starting point for future investigations of *L. monocytogenes* regulatory networks, including those that regulate primary metabolic functions under different stress conditions.

### 4.1. Transcripts of Genes Involved in Stress Response Are Among the Most Abundant under Stationary Phase

*L. monocytogenes* cells grown to stationary phase are known to express active forms of all alternative σ factors [12]. Stationary phase represents a nutrient depletion stress but also induces thiol and oxidative stress [34,35]. In addition, we observed a slight, but significant decline in the pH of the cultures with mutant strains in comparison to the cultures with the parent strain. As *L. monocytogenes* seems to be able to maintain the intracellular pH close to eight independent of the external pH in the range of 5–9 [70,71], it is unclear whether the intracellular pH of the mutant strains is affected by the lower pH observed in the media. The genes with the highest transcript levels in the parent strain and the strain expressing none of the alternative σ factors (ΔBCHL strain) were *csbD* and *fri*, respectively. These two genes have been previously reported to be involved in stress response. Moreover, *csbD*, which was also the highest transcribed gene in the ΔCHL strain (which expresses σ^A^ and σ^B^ only), is a general stress-response gene in *Bacillus* and, in that organism, has been shown to play a role in survival under salt and low temperature stress [72]. Ferritin, which is encoded by *fri*, is an iron-storage protein [39] that binds to iron, to prevent the interaction between ferrous iron and oxygen, which leads to the production of reactive oxygen species. The iron-loaded form of some ferritin proteins can bind to DNA and protect it under oxidative-stress conditions [40]. Moreover, *fri* has been reported to show evidence of growth-phase-dependent transcription [73,74], and is repressed by the ferric uptake repressor Fur [73] and peroxide stress-response repressor PerR [39]. Overall, these findings support that alternative σ factor dependent and independent stress-response genes play an important role for *L. monocytogenes* survival in stationary phase.

### 4.2. σ^B^ Has Broad Effects on Multiple Pathways That Can Be Linked to Stress Response

While we identified that a large number of different pathways are positively regulated by σ^B^ (Figure 6), some of these have previously been reported. Below, we briefly discuss three major pathways/regulatory circuits for which our data provide new evidence for σ^B^-dependent regulation, including (i) acid stress and (ii) oxidative stress resistance pathways, and (iii) sugar transport associated with virulence regulation. Importantly, our data also further confirm possible mechanistic linkages by which σ^B^ modulates PrfA activity and hence expression of *L. monocytogenes* virulence genes.

Genes involved in acid stress presented some of the highest FC among σ^B^-dependent genes. For example, the highest FC due to σ^B^ was for *gabD* (FC = 5201, which encodes a succinate–semialdehyde dehydrogenase that degrades succinate semialdehyde into succinate; succinate can be used in *L. monocytogenes* incomplete TCA cycle [75,76] to generate energy. Importantly, a *gabD* deletion mutant previously showed enhanced sensitivity to acid stress comparable to that of a *sigB* deletion mutant [20], and the succinate–semialdehyde dehydrogenase activity encoded by this gene is linked to the GABA acid resistance pathway. Moreover, *gadD3*, a σ^B^-dependent gene, which showed a ~75 FC between the parent and the ΔCHL strains, is another key gene in this pathway; it encodes an enzyme that converts glutamate and a hydrogen proton into carbon dioxide and GABA (γ-aminobutyrate), which alleviates acid stress. A GABA-aminotransferase (ArgD) [77] can then convert GABA into succinate semialdehyde, linking the reactions carried out by the two σ^B^-dependent enzymes, GabD and GadD3. This reaction is important, as GABA is a toxic compound and has been shown to accumulate intracellularly under acid-stress conditions, presumably due to the fact that the reaction catalyzed by GadD3 utilizes an H^+^ therefore increasing the intracellular pH. In addition to ArgD mediated conversion of GABA into succinate semialdehyde, *L. monocytogenes* also encodes an antiporter mechanism carried out by GadT1 and GadT2 that utilizes intracellular GABA for acquisition of extracellular glutamate, providing another mechanism for GABA removal from the cell. The high FCs identified here for *gabD* suggest that GABA breakdown to succinate was a key mechanism for GABA detoxification during the acid stress imposed in the stationary phase cells.

In terms of oxidative stress, σ^B^ induces the transcription of *msrA* [78] and *uspl-3* [49], which have been predicted (*msrA*) and shown (*uspl*-*3*) to be involved in counterattacking this type of stress. The glutathione reductase encoding gene (*lmo1433*) was also highly differentially expressed in the presence of σ^B^ (FC = 215 [ΔCHL/ΔBCHL]; as also previously shown [24]). Glutathione (GSH) is a low-molecular-weight thiol that plays major roles in maintaining the cytoplasm redox balance under thiol-stress conditions and in detoxification of reactive oxygen and nitrogen species [79]. Under oxidative-stress conditions, GSH protects proteins from over-oxidation by glutathionylation of cysteine residues. Re-activation of glutathionylated proteins by glutaredoxins leads to the formation of oxidized di-glutathione G-S-S-G [80]. G-S-S-G is non-functional and has to be reduced by the action of another enzyme, the GSH reductase [79], which is encoded by the σ^B^-regulated *lmo1433* [24]. As GSH has also been shown to be a cofactor required for full activation of PrfA [81], the regulation of *lmo1433* by σ^B^ may represent a mechanism by which σ^B^ indirectly contributes to regulating PrfA activity and hence transcription of the virulence genes in the PrfA regulon [23].

Sugar transporters were also found to be under σ^B^ regulation. Specifically, *glcU-1*, which encodes for a glucose permease and *mpoABCD*, which encode for a mannose/glucose PTS, was overexpressed in the mutant expressing σ^B^. Interestingly, Mpo has been hypothesized to be involved in PrfA activation upon glucose availability [82,83]. This hypothesis predicts that transport of glucose through Mpo, which generates intracellular glucose-6-phosphate, induces the activation of ManR, a positive regulator of the *man* operon, another mannose/glucose PTS encoding operon. The Man PTS would then repress PrfA, due to increased intracellular glucose levels provided by the Man PTS, resulting in decreased expression of the major virulence genes [82]. Consistent with this, a microarray study with *sigB* and *prfA* single and double mutants showed that expression of σ^B^ down-regulates expression of the PrfA regulon [26].

### 4.3. σ^L^ and σ^H^ Show Positive Regulation of More Narrow and Targeted Pathways Than the σ^B^ Regulon

Our data further support that σ^L^ positively regulates genes involved in pathways related to sugar transport and metabolism, as previously seen in other studies [12,48,84]. The 35 genes positively regulated by σ^L^ include genes encoding (i) PTS proteins that facilitate transport of sugars other than glucose (e.g., fructose, galactitol, and lactose; Stoll and Goebel, 2010), (ii) enzymes used to convert these sugars into glucose-6-phosphate (G6P), and (iii) enzymes of the non-oxidative branch of the Pentose Phosphate Pathway, which uses ribulose-6-phosphate (Ru6P) to generate the intermediates ribose-6-phosphate (R6P). R6P is then used for nucleotide biosynthesis, and biosynthesis of glyceraldehyde-3-phosphate (GAP) and fructose-6-phosphate (F6P) that can be consumed by glycolysis to generate energy or can be recycled by gluconeogenesis to generate G6P (Figure 6). Thus, our results confirm the previously observed role of σ^L^ in regulating *L. monocytogenes*’ energy metabolism [12,84].

Although σ^H^ was found to positively regulate a similar number of genes as σ^L^, the role of σ^H^ during starvation stress is not clear. Many genes identified as positively regulated by σ^H^ are annotated as having “unknown functions”. Moreover, σ^H^-dependent genes with known function include genes involved in regulation of competence, as also previously observed [14]. Although *L. monocytogenes* has been shown not to be naturally competent, a study has shown that, during intracellular growth in activated macrophages, genes involved in competence are expressed and are necessary for efficient escape from the macrophage phagosome [85]. This same study showed that expression of these competence genes depends on the expression of ComK, the master regulator of competence in *B. subtilis*. Interestingly, *comK* is interrupted by a prophage in *L. monocytogenes* 10403S strain, suggesting that the competence genes up-regulated by σ^H^ here (i.e., *comEA* and *coiA*) are not co-regulated by ComK in 10403S.

### 4.4. Epistatic and Additive Interactions among the Alternative σ Factors Regulate a Number of Genes and Functions, Including the Gene Encoding the Listeria Adhesion Protein (LAP), Involved in Listeria Virulence

Several genes showed patterns of transcript abundance, suggesting epistatic effects among the alternative σ factors. Most of the genes showing a negative epistatic effect (124 out of 191 genes) showed FC > 5 in the ΔBCL strain, suggesting that genes directly or indirectly positively regulated by σ^H^ are also indirectly repressed by other alternative sigma factors in *L. monocytogenes*. It has been previously suggested that σ^H^ and σ^L^ mediate the PTS-dependent growth of *L. monocytogenes* through complex transcriptional interactions and fine-tuning of the expression of specific *pts* genes [48]. Among those genes, *lmo1634* encodes the *Listeria* adhesion protein (LAP), which has been shown to play a role in *L. monocytogenes*’ virulence by promoting paracellular translocation of the bacterial pathogen through intestinal epithelial cell junctions [58]. In our study, *lmo1634* showed an FC > 50 in the mutant strain expressing σ^H^, and FC > 4 in each of the other mutant strains expressing σ^B^, σ^C^, or σ^L^, but an FC < 3 in the parent strain. This pattern suggests that *lmo1634* may be up-regulated by σ^H^, but this up-regulation is decreased when the other alternative σ factors are also expressed. Previous studies have shown that *lmo1634* is up-regulated in nutrient-depleted [86], high-temperature (42 °C) [87], and anaerobic [57] conditions. A previous study of the *L. monocytogenes* alternative σ factors regulons using single gene mutants (i.e., mutants with knock-out deletions in one of the four alternative σ factor genes at a time) did not identify *lmo1634* as being up-regulated by any of the four alternative σ factors [12], supporting the importance of the approach used here.

## 5. Conclusions

Alternative σ factors play a major role during *L. monocytogenes* adaptation to stationary phase. Genes up-regulated by alternative σ factors during stationary phase seem to provide the bacterium with a wider range of usable resources for energy supply concomitantly with a defense against other stresses that may occur in the stationary phase, such as acid stress, oxidative stress, and osmotic stress. Here, we provided comprehensive analyses of the alternative σ factor regulons in *L. monocytogenes* and showed that these regulons include considerable overlaps, creating a regulatory network that can fine-tune gene expression of *L. monocytogenes* in different environmental conditions, including those encountered in the host. Moreover, for the manual annotation of transcription start sites, 5′ UTR and promoter regions using RNA-Seq data provide valuable information to researchers studying *L. monocytogenes* and specifically to those using the laboratory strain 10403S; these data are publicly available at the eCommons database (https://doi.org/10.7298/0mjr-6c90). In addition, the RNA-Seq data provided through this study provide a rich public resource that can be used to further study interactions between *L. monocytogenes* σ factors and will support further discovery of epistatic and additive interactions among the alternative σ factors.

## Figures and Tables

**Table 1:** Strains used in this study.

Strain	Genotype	Sigma Factors Expressed
10403S	Wild-type	σ^A^, σ^B^, σ^C^, σ^H^, σ^L^
FSL ^a^ C3-139	Δ*sigC*, Δ*sigH*, Δ*sigL*	σ^A^, σ^B^
FSL C3-138	Δ*sigB*, Δ*sigH*, Δ*sigL*	σ^A^, σ^C^
FSL C3-137	Δ*sigB*, Δ*sigC*, Δ*sigL*	σ^A^, σ^H^
FSL C3-128	Δ*sigB*, Δ*sigC*, Δ*sigH*	σ^A^, σ^L^
FSL C3-135	Δ*sigB*, Δ*sigC*, Δ*sigH*, Δ*sigL*	σ^A^

^a^ FSL = Food Safety Lab.

**Table 2:** RNA-Seq summary data.

Strain	Replicate	Directional Run	Number of Mapped Reads (%) ^a^	Coefficient of Variation ^b^
10403S	1	Yes	7666449 (89.5)	0.433
10403S	2	Yes	10339693 (46.8)	
FSL C3-139	1	Yes	15386572 (69.2)	0.451
FSL C3-139	2	No	7904766 (88.4)	
FSL C3-138	1	Yes	13145762 (69.1)	0.447
FSL C3-138	2	No	7691707 (88.2)	
FSL C3-137	1	Yes	16116345 (78.7)	0.161
FSL C3-137	2	Yes	15503451 (65.6)	
FSL C3-128	1	Yes	14388951 (78.4)	0.165
FSL C3-128	2	Yes	16603748 (68.6)	
FSL C3-135	1	Yes	9988314 (63.2)	0.705
FSL C3-135	2	No	13704016 (97.4)	

^a^ Percentage of the total number of sequenced reads that mapped to the strain 10403S genome. ^b^ Coefficient of variation between the two replicates of the same strain.

**Table 3:** Complex expression patterns involving alternative σ factors.

**Genes with Lower Transcript Levels in the Parent Strain (10403S) in Comparison to the ΔBCHL Strain (FC < 0.2) and Higher Transcript Levels in at least One of ΔCHL, ΔBHL, ΔBCL, and ΔBCH Strains in Comparison to the ΔBCHL Strain (FC > 5.0) ^a^.**							
**10403S Locus**	**EGD-e Locus Name**	**10403S FC**	**σ^L^ FC**	**σ^H^ FC**	**σ^C^ FC**	**σ^B^ FC**	**Function**
LMRG_00198	lmo0517	0.08	0.09	11.91	2.55	2.56	similar to phosphoglycerate mutase
LMRG_02255	lmo0832	0.00	1.50	3.70	4.89	5.20	similar to transposase
LMRG_00881	lmo1429	0.14	0.07	0.65	7.06	4.01	similar to unknown proteins
LMRG_01244	lmo2093	0.20	0.68	8.88	10.02	1.98	unknown
LMRG_01245	lmo2094	0.07	0.31	4.44	10.25	1.10	similar to L-fuculose-phosphate aldolase
LMRG_01247	lmo2096	0.09	0.21	3.94	13.22	1.27	similar to PTS system galactitol-specific enzyme IIC component
LMRG_01598	lmo2234	0.00	0.48	15.63	0.00	0.52	similar to unknown proteins
LMRG_02720	lmo2375	0.00	0.59	3.21	7.33	8.58	unknown
LMRG_01751	lmo2497	0.00	0.35	1.85	3.44	15.13	similar to phosphate ABC transporter (permease protein)
**Genes with Mildly Lower Transcript Levels in the Parent Strain in Comparison to the ΔBCHL Strain (0.2 < FC < 0.5) and High FC (FC > 5.0) in at least one of the Triple Mutants Strain Expressing one of the ΔCHL, ΔBHL, ΔBCL, and ΔBCH Strains in Comparison to the ΔBCHL Strain (FC > 5.0) ^a^.**							
LMRG_00154	NoID	0.36	0.51	5.06	0.49	0.68	-
LMRG_02398	lmo0153	0.48	0.13	1.16	8.95	1.06	similar to a probable high-affinity zinc ABC transporter (Zn(II)-binding lipoprotein)
LMRG_00158	lmo0477	0.38	0.67	8.00	2.87	5.49	putative secreted protein
LMRG_00287	lmo0604	0.30	0.17	0.70	5.30	2.63	similar to *B. subtilis* YvlA protein
LMRG_00318	lmo0635	0.36	0.69	5.90	2.74	3.02	unknown
LMRG_00476	lmo0788	0.46	0.68	7.12	2.26	2.28	unknown
LMRG_01248	lmo2097	0.32	0.65	13.45	23.61	2.61	similar to PTS system galactitol-specific enzyme IIB component
LMRG_01970	lmo2726	0.29	0.32	5.45	2.73	2.74	similar to transcription regulators
LMRG_02138	NoID	0.39	0.72	5.13	1.04	0.97	-
					0.49	0.68	-
**Genes with High Positive Fold Change (FC > 5.0) ^a^ in at least Two of the ΔCHL, ΔBHL, ΔBCL, ΔBCH and Higher FC in the Parent Strain when Compared to the ΔBCHL Strain (Additive Effect).**							
LMRG_01216	lmo2066	32.40	4.61	15.27	11.18	13.46	unknown
LMRG_02207	lmo2662	40.53	32.08	11.56	2.18	14.62	similar to ribose 5-phosphate epimerase
LMRG_02208	lmo2663	94.76	68.20	22.11	3.04	24.13	similar to polyol dehydrogenase
LMRG_02209	lmo2664	59.73	44.42	16.19	2.36	24.58	similar to sorbitol dehydrogenase
LMRG_02210	lmo2665	43.67	33.93	15.14	2.60	18.64	similar to PTS system galactitol-specific enzyme IIC component
LMRG_02211	lmo2666	34.02	29.44	19.08	1.95	8.43	similar to PTS system galactitol-specific enzyme IIB component
LMRG_02212	lmo2667	35.69	26.23	18.80	1.57	8.67	similar to PTS system galactitol-specific enzyme IIA component
LMRG_02213	lmo2668	30.72	13.91	12.36	1.77	8.30	similar to transcriptional antiterminator (BglG family)

^a^ Fold Changes (FCs) > 5.0 are underlined.

## Data Availability

Coverage files and tables used in this study are publicly available at the eCommons database (https://doi.org/10.7298/0mjr-6c90).

## Supplementary Materials

The following are available online at https://www.mdpi.com/article/10.3390/pathogens10040411/s1. Figure S1: Growth curves for the parent strain (10403S) and the five mutant strains. (A) Log-transformed colony-forming units (CFU) over time. (B) Optical density (OD) over time. Figure S2: RNA-Seq detail of the LMRG_00091-LMRG_00095 operon (A,B) and the LMRG_00423-LMRG_00428 operon (C,D) encoding a fructose-specific PTS system and a putative D-allose-specific PTS system. (A,C) Overview of the whole operons. Horizontal arrows indicate the direction in which transcription occurs. Color-coded legends show the color corresponding to each strain in the coverage graph and the normalized average coverage for the entire region. (B,D) Detail of the promoter regions. Horizontal arrows show the indirection in which transcription occurs. The σ^A^-dependent promoters (i.e., σ^A^ -35 and -10 regions), transcription start (TS) sites and gene start (GS) sites are indicated. These two operons were highly expressed in the ΔBCH strain and they both present a putative regulatory motif (two conserved sequences separated by 16 nt indicated by a red “*”) just upstream the σ^A^-dependent -35 signal. Figure S3: RNA-Seq detail showing σ^B^-dependent expression of the σ^A^-dependent ncRNA Rli70 and the σB-dependent ncRNA Rli70-2 (both in yellow). Color-coded legends show the color corresponding to each strain in the coverage graph and the normalized average coverage for the two regions depicted by the two two-headed arrows shown above the coverage graph, which correspond to the Rli70 transcription region (left) and the Rli70-2-only transcription region (right). The σ^A^- and σ^B^-dependent promoters (i.e., σ^A^ -35 and -10 regions, and the σ^B^ -35 and -10 regions) and transcription start sites (TS) are indicated. Direction of transcription is indicated by horizontal arrows above rli70 and rli70-2. Figure S4: RNA-Seq detail of LMRG_02138-LMRG_02135 operon encoding CRISPR-associated proteins and showing a σ^B^-dependent anti-sense RNA overlapping the 5′ UTR of the operon. (A) Overview of the region. Graph shows the RNA-Seq coverage for each strain in the leading (top) and lagging (bottom) strands. Strains are color-coded as in the legend. Horizontal arrows indicate the direction in which transcription occurs. Coding genes are represented by blue bars while the noncoding RNA is represented by a yellow bar. The white bar represents the 5′ UTR region upstream the coding genes. (B) Detail of the noncoding RNA and overlapping region. Transcription start sites (TSSs), as well as the direction of transcription are indicated. The σ^A^-dependent promoters are shown. Color-coding is the same as for panel A. Figure S5: RNA-Seq detail of LMRG_02213-LMRG_02204 operon showing PTS genes and anti-sense RNA. (A) Detail of the LMRG_02213_LMRG_02204 operon promoter region. Horizontal one-headed arrows indicate the direction in which transcription occurs. Transcription start sites (TSSs), gene start codon (GS) and σ^A^-dependent -10 and -35 region (σ^A^ -10 and σ^A^ -35) are indicated. Coding genes are represented by blue bars while the 5′ UTR is represented by a white bar. The coverage graph shows the RNA-Seq coverage for each strain (color-coded). (B) Overview of the region showing the RNA-Seq coverage in both strands (direction of transcription is indicated by one-headed horizontal arrows). Strains are color-coded and the average normalized coverage values for each strain are shown within the legend for the region delimited by double-headed arrows. Values on top refer to the coverage in the leading strand while values on bottom refer to the coverages in the lagging strand. Noncoding anti-sense RNA is represented by a yellow bar, coding genes are represented by blue bars, and untranslated regions are represented by white bars. (C) Detail of the noncoding anti-sense RNA region. Coverages are color-coded and average normalized RNA-Seq coverage values are shown within the legend for the segment delimited by a double-headed arrow. Noncoding anti-sense RNA is represented by a yellow bar. A putative Rho-independent transcription terminator (TT) is represented by an orange bar. Coding genes are represented by blue bars and untranslated RNA is represented by white bars. The LMRG_02207 gene start codon (GS), and the σ^A^-dependent -10 and -35 regions (σ^A^ -10 and σ^A^ -35) are indicated. Figure S6. Hierarchical cluster tree showing heat map for all genes (σ^B^-dependent genes and σL-dependent genes involved in carbohydrate transport and metabolism are highlighted). (A) Heat map showing the Fold Changes (FCs) of every gene in the 10403S parent strain, ΔCHL, ΔBHL, ΔBCL, and ΔBCH strains relative to the ΔBCHL strain. Green indicates low FC and red indicated high FC. (B) Clusters (1–6) for selected groups highlighted in the heat map. Clusters 1–4 represent genes involved in carbohydrate metabolism and transport with high FC in the 10403S parent strain and the ΔBCH strain relative to the ΔBCHL strain. Clusters 5 and 6 represent σ^B^-dependent genes involved in various functions. Figure S7: RNA-Seq detail of LMRG_00334 (hypothetical protein) and the LMRG_00335-LMRG_00338 operon (encoding the magnesium and cobalt transporter CorA, two putative transcription regulators of the GntR family, and a phage infection protein) showing long σ^B^-dependent overlapping 5′ UTR regions. Horizontal one-headed arrows indicate the direction in which transcription occurs. Transcription start sites (TSSs), gene start codon (GS) and σ^A^- and σ^B^-dependent -10 and -35 region (σ^A^ -10, σ^A^ -35, σ^B^ -10, and σ^B^ -35) are indicated. Coding genes are represented by blue bars while the 5′ UTRs are represented by white bars. The coverage graph shows the RNA-Seq coverage for each strain (color-coded) and the average normalized coverage values for each strain are shown within the legend for the region delimited by double-headed arrows. Values on top refer to the coverage in the leading strand while values on bottom refer to the coverages in the lagging strand. Two putative Rho-independent transcription terminator (TT) are represented by red bars. Table S1: Cluster analysis by Fold Change. Table S2: Genes up-regulated by alternative sigma factors. Table S3: Genes down-regulated by alternative sigma factors. Table S4: Genes with divergent Fold Changes in 10403S and triple mutants. Table S5: Gene ontology enrichment analysis of up- and down-regulated genes. Table S6: Noncoding RNAs.
