# Supplementary material for: Alternative σ Factors Regulate Overlapping as Well as Distinct Stress Response and Metabolic Functions in Listeria monocytogenes under Stationary Phase Stress Condition
Source: Pathogens. 2021 Apr 1;10(4):411. doi: 10.3390/pathogens10040411 (PMC8066629; doi:10.3390/pathogens10040411)
Supplement: Supplementary file 1 [file pathogens-10-00411-s001.zip › rho10(02-22-2021)ST5.docx]

Table S5: Gene Ontology (GO) enrichment analysis of up- and down-regulated genes.

|  | **GO category** | **Name** | **Category** | **P-value** | **Q-value^a^** |
| --- | --- | --- | --- | --- | --- |
| σ^B^ positively regulated | | | | | |
|  | GO:0015418 | quaternary-ammonium-compound-transporting ATPase activity | molecular function | 9.72E-05 | 8.01E-02 |
|  | GO:0008509 | anion transmembrane transporter activity | molecular function | 2.83E-03 | 5.27E-01 |
|  | GO:0016811 | hydrolase activity, acting on carbon-nitrogen (but not peptide) bonds, in linear amides | molecular function | 3.46E-03 | 5.27E-01 |
|  | GO:0006820 | anion transport | biological process | 3.90E-03 | 5.27E-01 |
|  | GO:0015698 | inorganic anion transport | biological process | 3.90E-03 | 5.27E-01 |
|  | GO:0005618 | cell wall | cellular component | 4.48E-03 | 5.27E-01 |
|  | GO:0030312 | external encapsulating structure | cellular component | 4.48E-03 | 5.27E-01 |
|  | GO:0016810 | hydrolase activity, acting on carbon-nitrogen (but not peptide) bonds | molecular function | 6.06E-03 | 6.24E-01 |
|  | GO:0005315 | inorganic phosphate transmembrane transporter activity | molecular function | 1.31E-02 | 1.00E+00 |
|  | GO:0015114 | phosphate transmembrane transporter activity | molecular function | 1.78E-02 | 1.00E+00 |
|  | GO:0009986 | cell surface | cellular component | 2.26E-02 | 1.00E+00 |
|  | GO:0006817 | phosphate transport | biological process | 2.33E-02 | 1.00E+00 |
|  | GO:0016491 | oxidoreductase activity | molecular function | 2.68E-02 | 1.00E+00 |
|  | GO:0006041 | glucosamine metabolic process | biological process | 2.94E-02 | 1.00E+00 |
|  | GO:0006044 | N-acetylglucosamine metabolic process | biological process | 2.94E-02 | 1.00E+00 |
|  | GO:0044275 | cellular carbohydrate catabolic process | biological process | 4.14E-02 | 1.00E+00 |
| σ^L^ positively regulated | | | | | |
|  | GO:0015144 | carbohydrate transmembrane transporter activity | molecular function | 1.48E-06 | 5.53E-04 |
|  | GO:0009401 | phosphoenolpyruvate-dependent sugar phosphotransferase system | biological process | 1.58E-06 | 5.53E-04 |
|  | GO:0008982 | protein-N(PI)-phosphohistidine-sugar phosphotransferase activity | molecular function | 2.04E-06 | 5.53E-04 |
|  | GO:0008643 | carbohydrate transport | biological process | 4.39E-06 | 8.93E-04 |
|  | GO:0016772 | transferase activity, transferring phosphorus-containing groups | molecular function | 8.56E-06 | 9.92E-04 |
|  | GO:0005351 | sugar:hydrogen symporter activity | molecular function | 9.75E-06 | 9.92E-04 |
|  | GO:0005402 | cation:sugar symporter activity | molecular function | 9.75E-06 | 9.92E-04 |
|  | GO:0015295 | solute:hydrogen symporter activity | molecular function | 9.75E-06 | 9.92E-04 |
|  | GO:0015294 | solute:cation symporter activity | molecular function | 1.21E-05 | 1.09E-03 |
|  | GO:0015293 | symporter activity | molecular function | 1.48E-05 | 1.20E-03 |
|  | GO:0051119 | sugar transmembrane transporter activity | molecular function | 1.70E-05 | 1.26E-03 |
|  | GO:0016773 | phosphotransferase activity, alcohol group as acceptor | molecular function | 2.81E-05 | 1.85E-03 |
|  | GO:0007165 | signal transduction | biological process | 2.96E-05 | 1.85E-03 |
|  | GO:0007154 | cell communication | biological process | 7.24E-05 | 4.21E-03 |
|  | GO:0015291 | secondary active transmembrane transporter activity | molecular function | 1.88E-04 | 1.02E-02 |
|  | GO:0016740 | transferase activity | molecular function | 7.65E-04 | 3.89E-02 |
|  | GO:0008324 | cation transmembrane transporter activity | molecular function | 8.17E-04 | 3.91E-02 |
|  | GO:0016301 | kinase activity | molecular function | 1.02E-03 | 4.18E-02 |
|  | GO:0022891 | substrate-specific transmembrane transporter activity | molecular function | 1.02E-03 | 4.18E-02 |
|  | GO:0022804 | active transmembrane transporter activity | molecular function | 1.03E-03 | 4.18E-02 |
|  | GO:0004553 | hydrolase activity, hydrolyzing O-glycosyl compounds | molecular function | 1.15E-03 | 4.47E-02 |
|  | GO:0005975 | carbohydrate metabolic process | biological process | 1.37E-03 | 5.02E-02 |
|  | GO:0003824 | catalytic activity | molecular function | 1.42E-03 | 5.02E-02 |
|  | GO:0022892 | substrate-specific transporter activity | molecular function | 1.55E-03 | 5.25E-02 |
|  | GO:0050794 | regulation of cellular process | biological process | 1.68E-03 | 5.37E-02 |
|  | GO:0050789 | regulation of biological process | biological process | 1.72E-03 | 5.37E-02 |
|  | GO:0065007 | biological regulation | biological process | 2.42E-03 | 7.29E-02 |
|  | GO:0015075 | ion transmembrane transporter activity | molecular function | 2.57E-03 | 7.48E-02 |
|  | GO:0016798 | hydrolase activity, acting on glycosyl bonds | molecular function | 2.77E-03 | 7.77E-02 |
|  | GO:0016853 | isomerase activity | molecular function | 3.10E-03 | 8.41E-02 |
|  | GO:0022857 | transmembrane transporter activity | molecular function | 5.30E-03 | 1.39E-01 |
|  | GO:0005215 | transporter activity | molecular function | 1.00E-02 | 2.55E-01 |
|  | GO:0006098 | pentose-phosphate shunt | biological process | 1.15E-02 | 2.68E-01 |
|  | GO:0006739 | NADP metabolic process | biological process | 1.15E-02 | 2.68E-01 |
|  | GO:0006740 | NADPH regeneration | biological process | 1.15E-02 | 2.68E-01 |
|  | GO:0044275 | cellular carbohydrate catabolic process | biological process | 1.26E-02 | 2.84E-01 |
|  | GO:0006810 | transport | biological process | 1.63E-02 | 3.50E-01 |
|  | GO:0051234 | establishment of localization | biological process | 1.63E-02 | 3.50E-01 |
|  | GO:0016861 | intramolecular oxidoreductase activity, interconverting aldoses and ketoses | molecular function | 1.88E-02 | 3.92E-01 |
|  | GO:0044265 | cellular macromolecule catabolic process | biological process | 2.01E-02 | 4.08E-01 |
|  | GO:0006769 | nicotinamide metabolic process | biological process | 2.07E-02 | 4.11E-01 |
|  | GO:0016052 | carbohydrate catabolic process | biological process | 2.19E-02 | 4.17E-01 |
|  | GO:0016860 | intramolecular oxidoreductase activity | molecular function | 2.26E-02 | 4.17E-01 |
|  | GO:0009057 | macromolecule catabolic process | biological process | 2.28E-02 | 4.17E-01 |
|  | GO:0019362 | pyridine nucleotide metabolic process | biological process | 2.31E-02 | 4.17E-01 |
|  | GO:0044262 | cellular carbohydrate metabolic process | biological process | 2.50E-02 | 4.36E-01 |
|  | GO:0006733 | oxidoreduction coenzyme metabolic process | biological process | 2.52E-02 | 4.36E-01 |
|  | GO:0019318 | hexose metabolic process | biological process | 2.77E-02 | 4.70E-01 |
|  | GO:0005996 | monosaccharide metabolic process | biological process | 4.23E-02 | 6.91E-01 |
|  | GO:0006732 | coenzyme metabolic process | biological process | 4.24E-02 | 6.91E-01 |
| σ^L^ negatively regulated | | | | | |
|  | GO:0009058 | biosynthetic process | biological process | 6.63E-10 | 4.82E-07 |
|  | GO:0003735 | structural constituent of ribosome | molecular function | 5.91E-09 | 1.73E-06 |
|  | GO:0005198 | structural molecule activity | molecular function | 7.13E-09 | 1.73E-06 |
|  | GO:0030529 | ribonucleoprotein complex | cellular component | 9.61E-09 | 1.75E-06 |
|  | GO:0044249 | cellular biosynthetic process | biological process | 1.39E-08 | 2.02E-06 |
|  | GO:0006412 | translation | biological process | 2.59E-08 | 3.14E-06 |
|  | GO:0009059 | macromolecule biosynthetic process | biological process | 2.67E-07 | 2.77E-05 |
|  | GO:0043284 | biopolymer biosynthetic process | biological process | 3.15E-07 | 2.86E-05 |
|  | GO:0034645 | cellular macromolecule biosynthetic process | biological process | 5.74E-07 | 4.63E-05 |
|  | GO:0034961 | cellular biopolymer biosynthetic process | biological process | 9.81E-07 | 7.13E-05 |
|  | GO:0044444 | cytoplasmic part | cellular component | 3.14E-06 | 2.07E-04 |
|  | GO:0019843 | rRNA binding | molecular function | 6.35E-06 | 3.85E-04 |
|  | GO:0032991 | macromolecular complex | cellular component | 9.36E-06 | 5.23E-04 |
|  | GO:0044255 | cellular lipid metabolic process | biological process | 1.07E-05 | 5.55E-04 |
|  | GO:0005840 | ribosome | cellular component | 1.96E-05 | 9.50E-04 |
|  | GO:0043283 | biopolymer metabolic process | biological process | 2.29E-05 | 1.04E-03 |
|  | GO:0044464 | cell part | cellular component | 2.59E-05 | 1.05E-03 |
|  | GO:0016020 | membrane | cellular component | 2.60E-05 | 1.05E-03 |
|  | GO:0008610 | lipid biosynthetic process | biological process | 3.97E-05 | 1.52E-03 |
|  | GO:0034960 | cellular biopolymer metabolic process | biological process | 4.40E-05 | 1.54E-03 |
|  | GO:0005575 | cellular component | cellular component | 4.45E-05 | 1.54E-03 |
|  | GO:0033279 | ribosomal subunit | cellular component | 8.19E-05 | 2.71E-03 |
|  | GO:0051188 | cofactor biosynthetic process | biological process | 9.98E-05 | 3.15E-03 |
|  | GO:0006629 | lipid metabolic process | biological process | 2.02E-04 | 6.10E-03 |
|  | GO:0019861 | flagellum | cellular component | 2.31E-04 | 6.42E-03 |
|  | GO:0042995 | cell projection | cellular component | 2.31E-04 | 6.42E-03 |
|  | GO:0043228 | non-membrane-bounded organelle | cellular component | 2.49E-04 | 6.42E-03 |
|  | GO:0043232 | intracellular non-membrane-bounded organelle | cellular component | 2.49E-04 | 6.42E-03 |
|  | GO:0051128 | regulation of cellular component organization and biogenesis | biological process | 2.56E-04 | 6.42E-03 |
|  | GO:0000270 | peptidoglycan metabolic process | biological process | 4.53E-04 | 1.01E-02 |
|  | GO:0016874 | ligase activity | molecular function | 4.72E-04 | 1.01E-02 |
|  | GO:0003723 | RNA binding | molecular function | 4.73E-04 | 1.01E-02 |
|  | GO:0008360 | regulation of cell shape | biological process | 5.00E-04 | 1.01E-02 |
|  | GO:0022603 | regulation of anatomical structure morphogenesis | biological process | 5.00E-04 | 1.01E-02 |
|  | GO:0022604 | regulation of cell morphogenesis | biological process | 5.00E-04 | 1.01E-02 |
|  | GO:0050793 | regulation of developmental process | biological process | 5.00E-04 | 1.01E-02 |
|  | GO:0044237 | cellular metabolic process | biological process | 5.97E-04 | 1.12E-02 |
|  | GO:0033013 | tetrapyrrole metabolic process | biological process | 6.02E-04 | 1.12E-02 |
|  | GO:0033014 | tetrapyrrole biosynthetic process | biological process | 6.02E-04 | 1.12E-02 |
|  | GO:0043226 | organelle | cellular component | 8.16E-04 | 1.45E-02 |
|  | GO:0043229 | intracellular organelle | cellular component | 8.16E-04 | 1.45E-02 |
|  | GO:0044238 | primary metabolic process | biological process | 1.04E-03 | 1.80E-02 |
|  | GO:0006778 | porphyrin metabolic process | biological process | 1.11E-03 | 1.83E-02 |
|  | GO:0006779 | porphyrin biosynthetic process | biological process | 1.11E-03 | 1.83E-02 |
|  | GO:0008152 | metabolic process | biological process | 1.17E-03 | 1.88E-02 |
|  | GO:0044422 | organelle part | cellular component | 1.36E-03 | 2.11E-02 |
|  | GO:0044446 | intracellular organelle part | cellular component | 1.36E-03 | 2.11E-02 |
|  | GO:0016051 | carbohydrate biosynthetic process | biological process | 1.86E-03 | 2.82E-02 |
|  | GO:0044267 | cellular protein metabolic process | biological process | 2.12E-03 | 3.14E-02 |
|  | GO:0009225 | nucleotide-sugar metabolic process | biological process | 2.56E-03 | 3.72E-02 |
|  | GO:0051301 | cell division | biological process | 2.92E-03 | 4.16E-02 |
|  | GO:0016053 | organic acid biosynthetic process | biological process | 3.25E-03 | 4.46E-02 |
|  | GO:0019538 | protein metabolic process | biological process | 3.25E-03 | 4.46E-02 |
|  | GO:0015934 | large ribosomal subunit | cellular component | 3.41E-03 | 4.59E-02 |
|  | GO:0046483 | heterocycle metabolic process | biological process | 4.35E-03 | 5.59E-02 |
|  | GO:0032553 | ribonucleotide binding | molecular function | 4.39E-03 | 5.59E-02 |
|  | GO:0032555 | purine ribonucleotide binding | molecular function | 4.39E-03 | 5.59E-02 |
|  | GO:0016780 | phosphotransferase activity, for other substituted phosphate groups | molecular function | 5.23E-03 | 6.56E-02 |
|  | GO:0009987 | cellular process | biological process | 5.96E-03 | 7.35E-02 |
|  | GO:0043170 | macromolecule metabolic process | biological process | 6.41E-03 | 7.76E-02 |
|  | GO:0009451 | RNA modification | biological process | 6.88E-03 | 8.19E-02 |
|  | GO:0005525 | GTP binding | molecular function | 7.40E-03 | 8.40E-02 |
|  | GO:0019001 | guanyl nucleotide binding | molecular function | 7.40E-03 | 8.40E-02 |
|  | GO:0032561 | guanyl ribonucleotide binding | molecular function | 7.40E-03 | 8.40E-02 |
|  | GO:0009108 | coenzyme biosynthetic process | biological process | 7.91E-03 | 8.85E-02 |
|  | GO:0006633 | fatty acid biosynthetic process | biological process | 8.65E-03 | 9.52E-02 |
|  | GO:0006631 | fatty acid metabolic process | biological process | 9.21E-03 | 9.78E-02 |
|  | GO:0005886 | plasma membrane | cellular component | 9.26E-03 | 9.78E-02 |
|  | GO:0004312 | fatty-acid synthase activity | molecular function | 9.29E-03 | 9.78E-02 |
|  | GO:0044260 | cellular macromolecule metabolic process | biological process | 1.11E-02 | 1.09E-01 |
|  | GO:0044424 | intracellular part | cellular component | 1.12E-02 | 1.09E-01 |
|  | GO:0008654 | phospholipid biosynthetic process | biological process | 1.13E-02 | 1.09E-01 |
|  | GO:0046467 | membrane lipid biosynthetic process | biological process | 1.13E-02 | 1.09E-01 |
|  | GO:0006643 | membrane lipid metabolic process | biological process | 1.14E-02 | 1.09E-01 |
|  | GO:0006644 | phospholipid metabolic process | biological process | 1.14E-02 | 1.09E-01 |
|  | GO:0015935 | small ribosomal subunit | cellular component | 1.14E-02 | 1.09E-01 |
|  | GO:0030955 | potassium ion binding | molecular function | 1.17E-02 | 1.09E-01 |
|  | GO:0031420 | alkali metal ion binding | molecular function | 1.17E-02 | 1.09E-01 |
|  | GO:0051186 | cofactor metabolic process | biological process | 1.26E-02 | 1.15E-01 |
|  | GO:0009235 | cobalamin metabolic process | biological process | 1.28E-02 | 1.15E-01 |
|  | GO:0009236 | cobalamin biosynthetic process | biological process | 1.28E-02 | 1.15E-01 |
|  | GO:0016879 | ligase activity, forming carbon-nitrogen bonds | molecular function | 1.40E-02 | 1.24E-01 |
|  | GO:0000049 | tRNA binding | molecular function | 1.43E-02 | 1.25E-01 |
|  | GO:0006396 | RNA processing | biological process | 1.48E-02 | 1.28E-01 |
|  | GO:0009252 | peptidoglycan biosynthetic process | biological process | 1.66E-02 | 1.42E-01 |
|  | GO:0017076 | purine nucleotide binding | molecular function | 1.69E-02 | 1.43E-01 |
|  | GO:0016070 | RNA metabolic process | biological process | 1.72E-02 | 1.44E-01 |
|  | GO:0007049 | cell cycle | biological process | 1.78E-02 | 1.45E-01 |
|  | GO:0003674 | molecular function | molecular function | 1.78E-02 | 1.45E-01 |
|  | GO:0016021 | integral to membrane | cellular component | 2.06E-02 | 1.64E-01 |
|  | GO:0031224 | intrinsic to membrane | cellular component | 2.06E-02 | 1.64E-01 |
|  | GO:0000166 | nucleotide binding | molecular function | 2.33E-02 | 1.84E-01 |
|  | GO:0055085 | transmembrane transport | biological process | 2.39E-02 | 1.87E-01 |
|  | GO:0046394 | carboxylic acid biosynthetic process | biological process | 2.51E-02 | 1.92E-01 |
|  | GO:0015849 | organic acid transport | biological process | 2.54E-02 | 1.92E-01 |
|  | GO:0046942 | carboxylic acid transport | biological process | 2.54E-02 | 1.92E-01 |
|  | GO:0044425 | membrane part | cellular component | 2.72E-02 | 2.03E-01 |
|  | GO:0034637 | cellular carbohydrate biosynthetic process | biological process | 2.75E-02 | 2.03E-01 |
|  | GO:0006721 | terpenoid metabolic process | biological process | 2.79E-02 | 2.03E-01 |
|  | GO:0016114 | terpenoid biosynthetic process | biological process | 2.79E-02 | 2.03E-01 |
|  | GO:0015837 | amine transport | biological process | 2.96E-02 | 2.13E-01 |
|  | GO:0000271 | polysaccharide biosynthetic process | biological process | 3.01E-02 | 2.15E-01 |
|  | GO:0006400 | tRNA modification | biological process | 3.09E-02 | 2.18E-01 |
|  | GO:0009253 | peptidoglycan catabolic process | biological process | 3.18E-02 | 2.22E-01 |
|  | GO:0043412 | biopolymer modification | biological process | 3.21E-02 | 2.22E-01 |
|  | GO:0034660 | ncRNA metabolic process | biological process | 3.25E-02 | 2.22E-01 |
|  | GO:0005524 | ATP binding | molecular function | 3.31E-02 | 2.23E-01 |
|  | GO:0032559 | adenyl ribonucleotide binding | molecular function | 3.31E-02 | 2.23E-01 |
|  | GO:0019205 | nucleobase, nucleoside, nucleotide kinase activity | molecular function | 3.46E-02 | 2.31E-01 |
|  | GO:0009110 | vitamin biosynthetic process | biological process | 3.53E-02 | 2.33E-01 |
|  | GO:0065008 | regulation of biological quality | biological process | 3.59E-02 | 2.35E-01 |
|  | GO:0003924 | GTPase activity | molecular function | 3.79E-02 | 2.46E-01 |
|  | GO:0034470 | ncRNA processing | biological process | 3.90E-02 | 2.51E-01 |
|  | GO:0016310 | phosphorylation | biological process | 4.20E-02 | 2.67E-01 |
|  | GO:0006865 | amino acid transport | biological process | 4.23E-02 | 2.67E-01 |
|  | GO:0019748 | secondary metabolic process | biological process | 4.48E-02 | 2.80E-01 |
|  | GO:0016740 | transferase activity | molecular function | 4.60E-02 | 2.85E-01 |
|  | GO:0009396 | folic acid and derivative biosynthetic process | biological process | 4.66E-02 | 2.86E-01 |
|  | GO:0005275 | amine transmembrane transporter activity | molecular function | 4.89E-02 | 2.86E-01 |
|  | GO:0005342 | organic acid transmembrane transporter activity | molecular function | 4.91E-02 | 2.86E-01 |
|  | GO:0046943 | carboxylic acid transmembrane transporter activity | molecular function | 4.91E-02 | 2.86E-01 |
|  | GO:0019349 | ribitol metabolic process | biological process | 4.94E-02 | 2.86E-01 |
|  | GO:0019350 | teichoic acid biosynthetic process | biological process | 4.94E-02 | 2.86E-01 |
|  | GO:0019519 | pentitol metabolic process | biological process | 4.94E-02 | 2.86E-01 |
|  | GO:0046374 | teichoic acid metabolic process | biological process | 4.94E-02 | 2.86E-01 |
|  | GO:0042278 | purine nucleoside metabolic process | biological process | 4.96E-02 | 2.86E-01 |
| σ^B^, σ^C^, σ^H^ positively regulated | | | | | |
|  | GO:0016070 | RNA metabolic process | biological process | 7.51E-03 | 1.00E+00 |
|  | GO:0008173 | RNA methyltransferase activity | molecular function | 8.14E-03 | 1.00E+00 |
|  | GO:0016833 | oxo-acid-lyase activity | molecular function | 1.18E-02 | 1.00E+00 |
|  | GO:0019104 | DNA N-glycosylase activity | molecular function | 1.68E-02 | 1.00E+00 |
|  | GO:0042430 | indole and derivative metabolic process | biological process | 1.81E-02 | 1.00E+00 |
|  | GO:0006586 | indolalkylamine metabolic process | biological process | 1.81E-02 | 1.00E+00 |
|  | GO:0006568 | tryptophan metabolic process | biological process | 1.81E-02 | 1.00E+00 |
|  | GO:0000162 | tryptophan biosynthetic process | biological process | 1.81E-02 | 1.00E+00 |
|  | GO:0042430 | indole and derivative metabolic process | biological process | 1.81E-02 | 1.00E+00 |
|  | GO:0042434 | indole derivative metabolic process | biological process | 1.81E-02 | 1.00E+00 |
|  | GO:0042435 | indole derivative biosynthetic process | biological process | 1.81E-02 | 1.00E+00 |
|  | GO:0046219 | indolalkylamine biosynthetic process | biological process | 2.30E-02 | 1.00E+00 |
|  | GO:0000162 | tryptophan biosynthetic process | biological process | 2.36E-02 | 1.00E+00 |
|  | GO:0006568 | tryptophan metabolic process | biological process | 2.36E-02 | 1.00E+00 |
|  | GO:0016799 | hydrolase activity, hydrolyzing N-glycosyl compounds | molecular function | 2.36E-02 | 1.00E+00 |
|  | GO:0006352 | transcription initiation | biological process | 2.36E-02 | 1.00E+00 |
|  | GO:0065004 | protein-DNA complex assembly | biological process | 2.46E-02 | 1.00E+00 |
|  | GO:0016987 | sigma factor activity | molecular function | 2.46E-02 | 1.00E+00 |
|  | GO:0016986 | transcription initiation factor activity | molecular function | 2.69E-02 | 1.00E+00 |
|  | GO:0034062 | RNA polymerase activity | molecular function | 2.77E-02 | 1.00E+00 |
|  | GO:0003899 | DNA-directed RNA polymerase activity | molecular function | 2.98E-02 | 1.00E+00 |
|  | GO:0009451 | RNA modification | biological process | 4.10E-02 | 1.00E+00 |
|  | GO:0003677 | DNA binding | molecular function | 4.10E-02 | 1.00E+00 |
|  | GO:0006284 | base-excision repair | biological process | 4.47E-02 | 1.00E+00 |
|  | GO:0042401 | biogenic amine biosynthetic process | biological process | 4.86E-02 | 1.00E+00 |
|  | GO:0042398 | amino acid derivative biosynthetic process | biological process | 7.51E-03 | 1.00E+00 |
|  | GO:0042401 | biogenic amine biosynthetic process | biological process | 8.14E-03 | 1.00E+00 |
|  | GO:0003676 | nucleic acid binding | molecular function | 1.18E-02 | 1.00E+00 |
|  | GO:0050896 | response to stimulus | biological process | 1.68E-02 | 1.00E+00 |

^a^ Q-values were calculated using the "qvalue" function implemented in R using the bootstrap option to estimate π_0_.
