# Supplementary figures and images for: Alternative σ Factors Regulate Overlapping as Well as Distinct Stress Response and Metabolic Functions in Listeria monocytogenes under Stationary Phase Stress Condition
Source: Pathogens. 2021 Apr 1;10(4):411. doi: 10.3390/pathogens10040411 (PMC8066629; doi:10.3390/pathogens10040411)

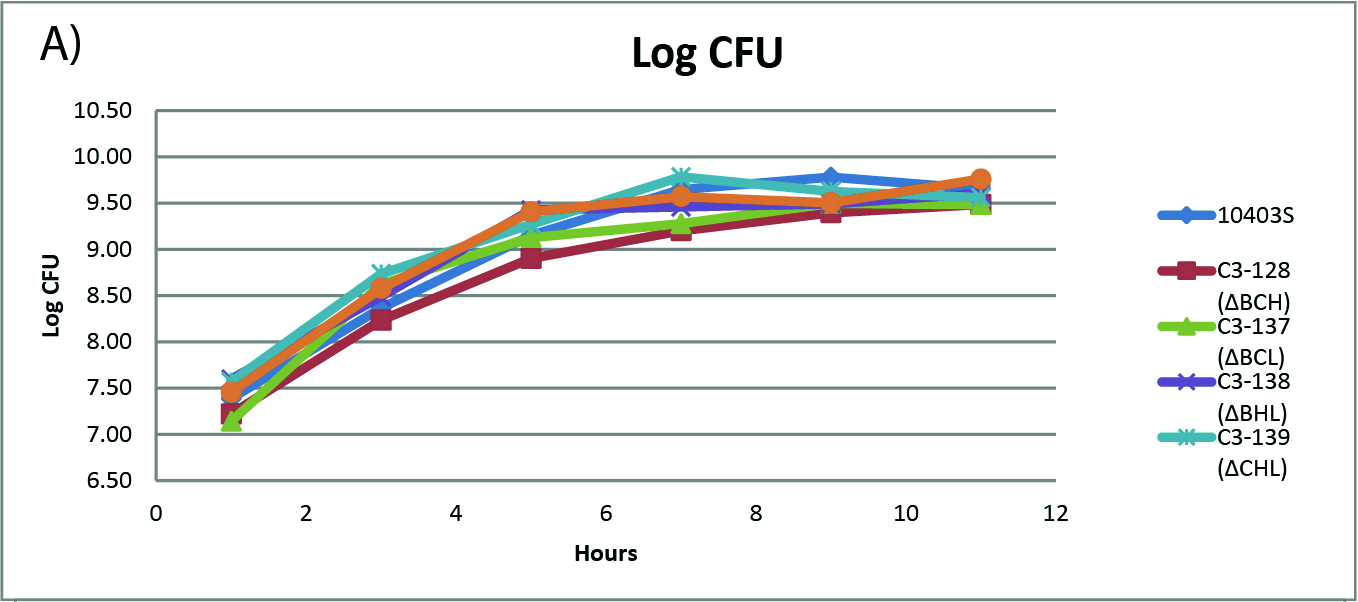

Supplement: Supplementary file 1 [file pathogens-10-00411-s001.zip › FigureS1_3-22-21_v2-02.tif]

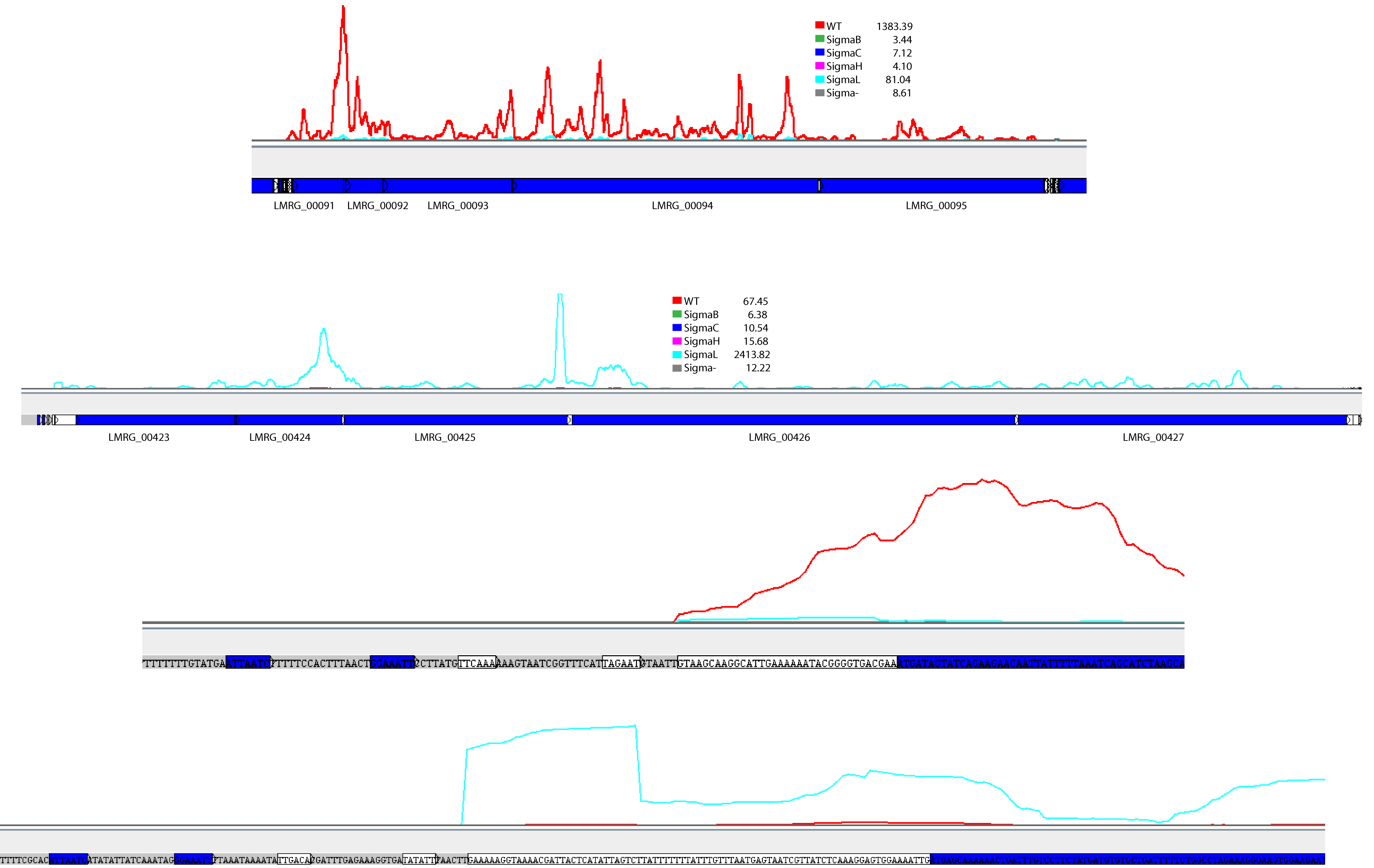

Supplement: Supplementary file 1 [file pathogens-10-00411-s001.zip › rho10(09-03-2012)SF2.tif]

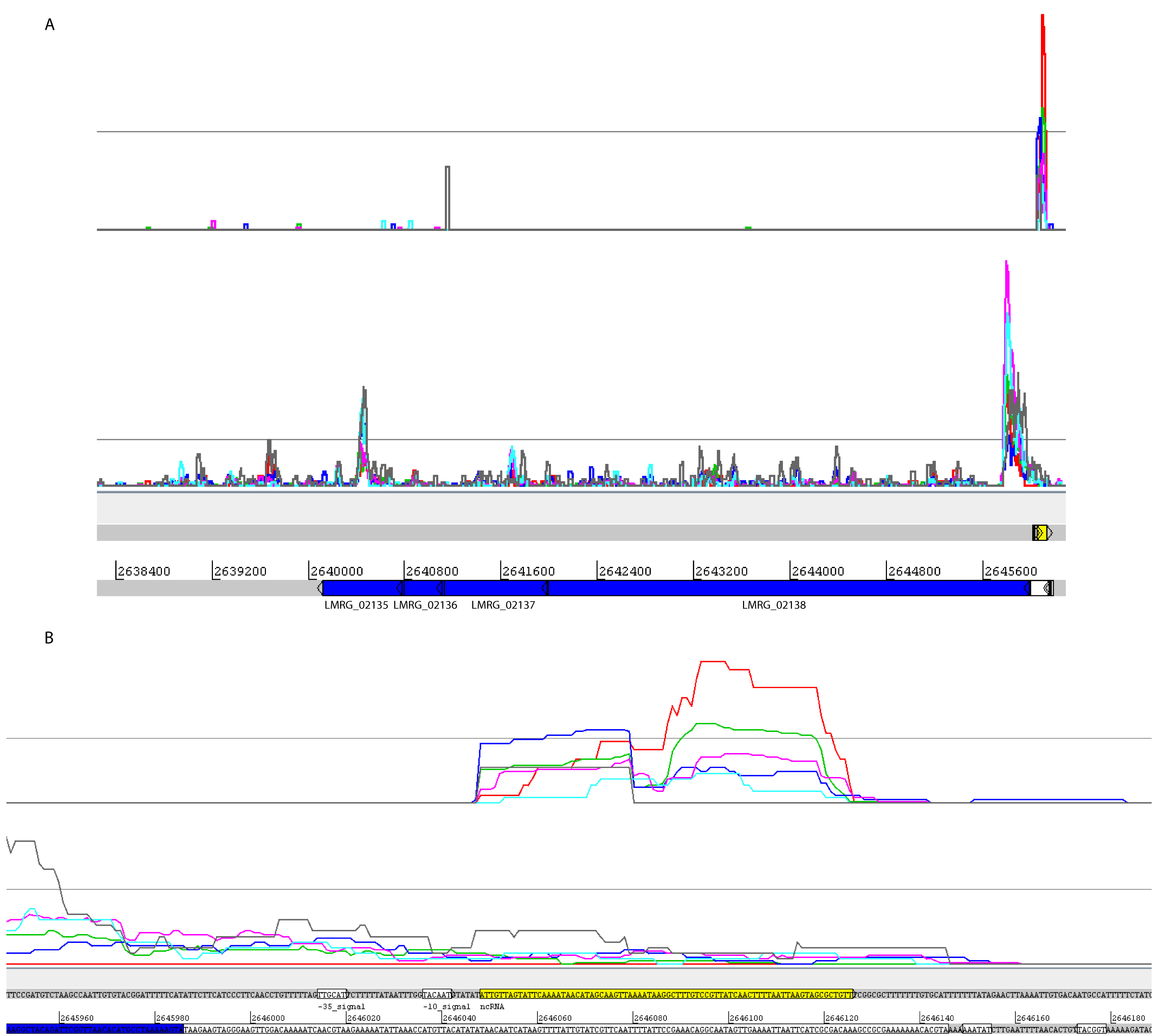

Supplement: Supplementary file 1 [file pathogens-10-00411-s001.zip › rho10(09-03-2012)SF4.tif]

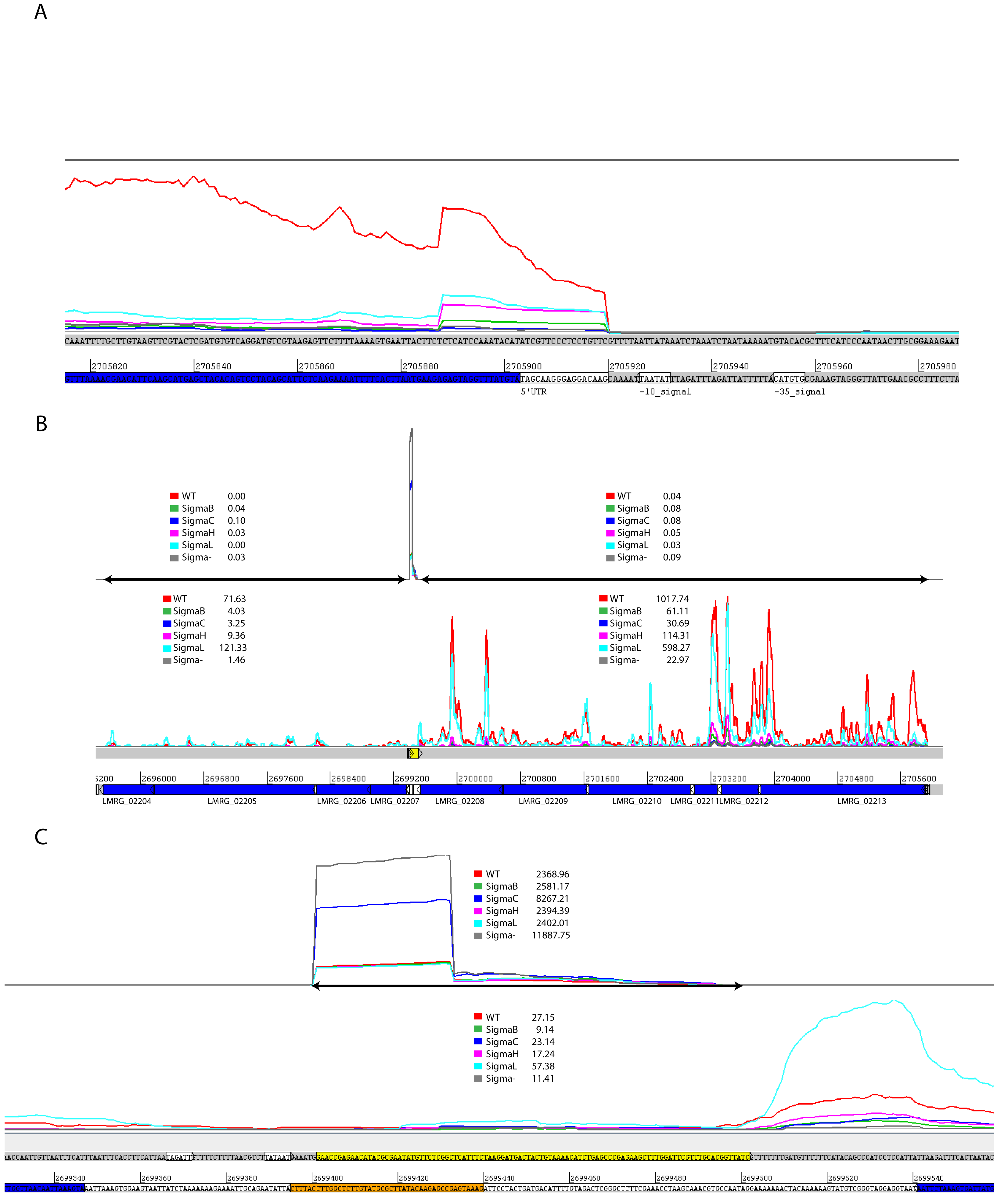

Supplement: Supplementary file 1 [file pathogens-10-00411-s001.zip › rho10(09-03-2012)SF5.tif]

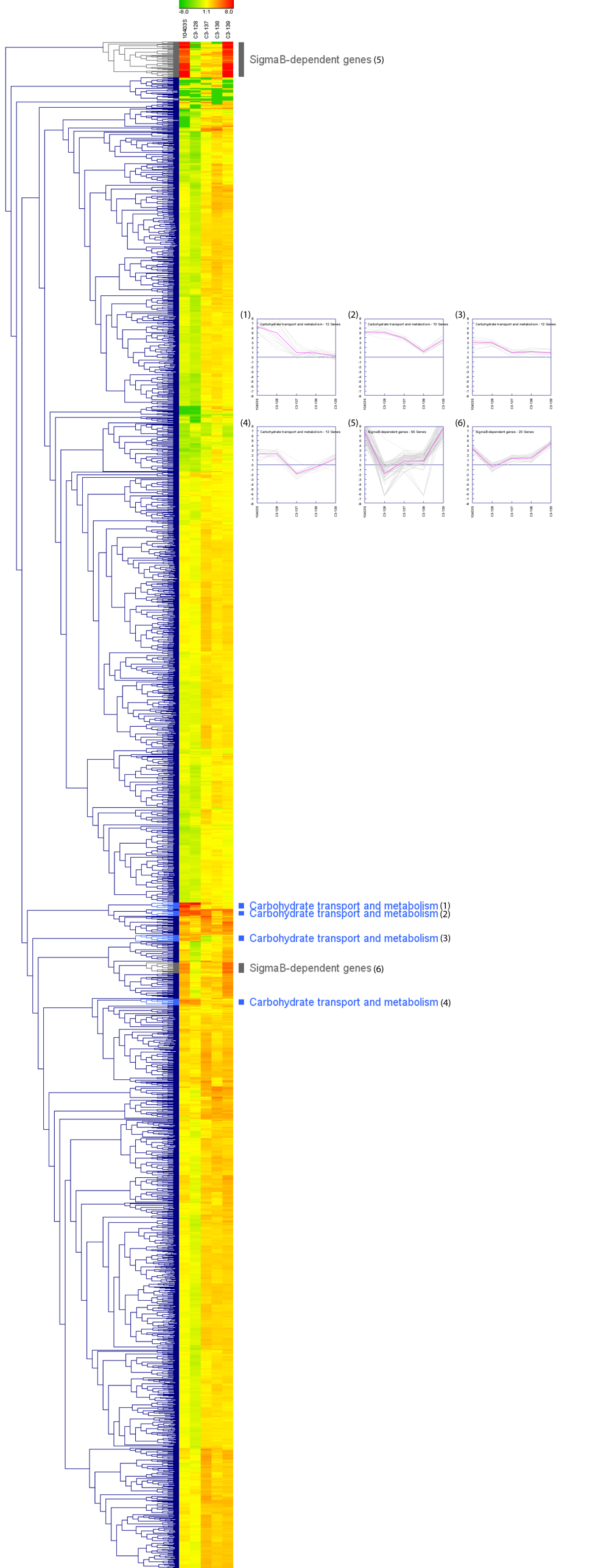

Supplement: Supplementary file 1 [file pathogens-10-00411-s001.zip › rho10(09-03-2012)SF6.tif]

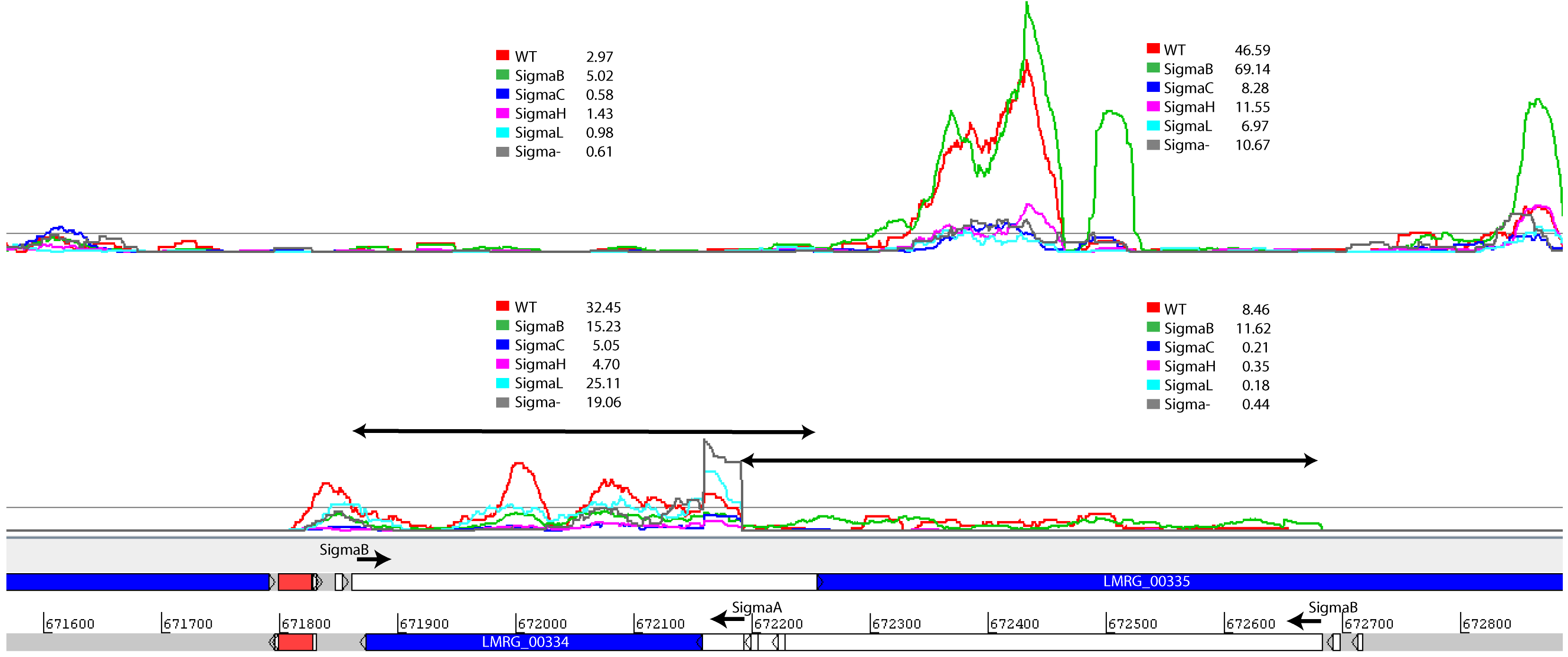

Supplement: Supplementary file 1 [file pathogens-10-00411-s001.zip › rho10(09-03-2012)SF7.tif]

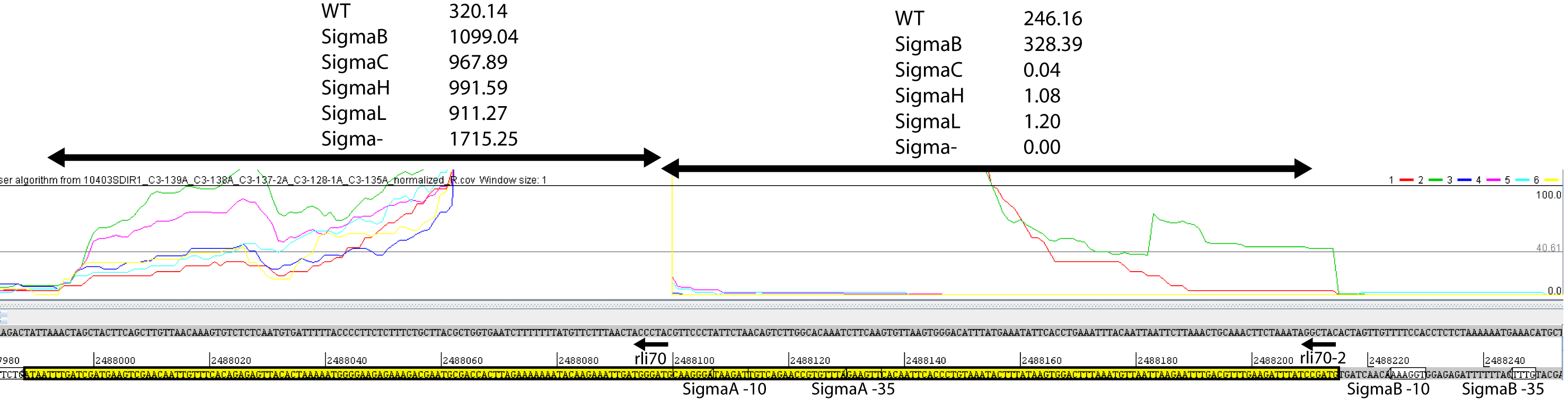

Supplement: Supplementary file 1 [file pathogens-10-00411-s001.zip › rho10(09-06-12)SF3.tif]
